# Integrated Transcriptomic and Metabolomic Analyses Reveal the Mechanisms Underlying Anthocyanin Coloration and Aroma Formation in Purple Fennel

**DOI:** 10.3389/fnut.2022.875360

**Published:** 2022-04-27

**Authors:** Yanjie Zhang, Qing Zhao, Youwei Feng, Yuanhang Dong, Tianjiao Zhang, Qiu Yang, Huihui Gu, Jinyong Huang, Yan Li

**Affiliations:** ^1^School of Agricultural Sciences, Zhengzhou University, Zhengzhou, China; ^2^The Center of Advanced Analysis and Gene Sequencing, Zhengzhou University, Zhengzhou, China

**Keywords:** anthocyanin, aroma, transcriptomics, metabolomics, fennel

## Abstract

The color and aroma are the significant traits of vegetables and fruits, but the metabolic and molecular mechanisms underlying anthocyanin accumulation and aroma formation remain almost unknown in fennel (*Anethum foeniculum* L.), which is a crucial vegetable crop and grown widely for aromatic leaves and bulbs. Here, ten major anthocyanins identified and quantified by ultra-high performance liquid chromatography coupled with quadrupole Orbitrap high-resolution mass spectrometry (UHPLC-Q-Orbitrap HRMS) were mainly responsible for the coloration of purple fennel leaf. With the application of GC-MS, it was found that the reduced volatile phenylpropanoids including isoeugenol, trans-isoeugenol, and apiol chiefly account for the characteristic aroma changes of the purple fennel. Moreover, the characteristic anthocyanin coloration and aroma formation in purple fennel were systematically studied with the integrated transcriptomics and metabolomics. The critical genes associated with the biosynthesis and regulation of anthocyanins and volatile phenylpropanoids were isolated and studied carefully in transiently transfected tobacco cells and transgenic tomato plants. Together with the results of UHPLC-Q-Orbitrap HRMS, RT-qPCR, and yeast two hybrid (Y2H), it is proved that the metabolic flux redirection of phenylpropanoid pathway primarily regulated by a functional MYB-bHLH-WD40 complex consisting of AfTT8, AfMYB7, and AfTTG1 accounts for the characteristic anthocyanin coloration and aroma formation in purple fennel leaf. The systematic understanding of the anthocyanin accumulation and aroma formation will assist in the improvement of fennel resource utilization and breeding.

## Introduction

Anthocyanins, an important subgroup of flavonoids with a basic carbon skeleton of C6-C3-C6, are widely distributed in plant kingdom and endow plant organs and tissues with eye-catching colors ranging from pale red to intensive blue (1, 2). Up to now, more than 600 kinds of anthocyanins identified and characterized from various plant species and even fungus. The type, content, and distribution of anthocyanins vary greatly, depending on genotypes, developmental stages, and environmental conditions (1, 2). Anthocyanins not only function importantly in pollination and seed dispersal, but also endow plants with strong environmental adaptability by protecting plant against various stresses, such as herbivore attack, pathogen infection, UV-B irradiation, drought stress, low temperature, nutrition deficiency, and osmotic stress (2–4). More important, lots of studies demonstrated that adequate intake of anthocyanins in diet can significantly reduce the risks of suffering from type 2 diabetes, atherosclerosis, cardiovascular diseases, neurodegenerative disorders, and cancer (5–9).

In phenylpropanoid metabolism, phenylalanine is the initial precursor of anthocyanins and other phenylpropanoids in the whole pathway. Anthocyanin biosynthesis starts directly with the condensation of one p-coumaroyl-CoA with three malonyl-CoA molecules, resulting in the generation of naringenin chalcone. In the subsequent biochemical reaction catalyzed by chalcone isomerase (CHI), flavone 3-hydroxylase (F3H), flavonoid 3',-hydroxylase (F3'H), dihydroflavonol reductase (DFR), and anthocyanidin synthase (ANS), naringenin chalcone is converted to colorful anthocyanidins (10). Finally, these anthocyanidin aglycones further undergo diverse kinds of glycosylation and acylation, to generate more stable anthocyanins for storage in plant cell (10). As a branch pathway of flavonoid biosynthesis, anthocyanin biosynthesis shares several conserve enzymes with other phenylpropanoids such as flavonols and flavones (10).

The phenylpropanoid metabolism is not only restricted to the biosynthesis of common flavonoids such as anthocyanins, but also generates various aromatic metabolites, such as phenylpropenes, coumarins, and hydrolysable tannins (11). These volatile metabolites not only function as pollinator attractants, but also as defense compounds protecting plant against various biotic attacks (12). With respect to phenylpropene volatiles such as eugenol and isoeugenol, they are produced through the phenylpropanoid pathway, such as the NADPH-dependent reduction of coniferyl acetate by eugenol synthase (EGS), which catalyzes the reductive replacement of the propenyl side chain (13). Methylation of the 4-hydroxy group of the above phenylpropenes by phenylpropene O-methyltransferases, a specific subfamily of methyltransferases, indicates the end of the phenylpropene pathway (12, 13). Excepting phenylpropane of phenylpropanoid pathway, other metabolic pathways can also produce volatile organic compounds (VOCs). Based on the biosynthetic origins, volatile terpenoids are mainly generated from mevalonate (MVA) or methylerythritol phosphate (MEP), whereas alcohols/aldehydes are generated from unsaturated fatty acids and amino acids (11, 12). Although many enzymes involved in the production of upstream metabolism of VOCs have been characterized in plants, most of the downstream enzymes of VOC pathways largely remain unknown (11, 12).

Fennel (*Anethum foeniculum* L.), an important aromatic plant of Apiaceae (Umbelliferae) family, is indigenous to the Mediterranean and nowadays cultivated worldwide (14, 15). Fennel seeds are mainly used as spice due to its characteristic and pleasant smell, which results from the accumulation of aromatic compounds, such as fenchone, limonene, and phenylpropenes from the phenylpropanoid pathway (16, 17). Additionally, the essential oils extracted from fennel seeds, generally recognized for their pharmacological activities, are widely used in the production of sweets, pastries, bread, and beverages and as natural preservative in food, cosmetic, and pharmaceutical products (16, 18). However, in many countries and regions, the edible leaf sheathes and tender leaves of fennel are fascinating autumn or winter vegetable particularly appreciated by the consumers for their aromatic flavor and fleshy texture. Since consumers usually assess the quality of vegetables on the basement of visual and olfactory perception (16), the identification and quantification of anthocyanins and VOCs in edible leaf are extremely important for fennel. Moreover, the regulatory genes responsible for the biosynthesis of these secondary metabolites also need to be revealed for further breeding.

Current studies mainly focused on the chemical compositions of fennel essential oils and their pharmacological activities (16, 17, 19). However, the molecular mechanisms underlying anthocyanin accumulation and aroma formation were seldomly studied and remain almost unknown in most aromatic herbs (20). In this study, we characterized and compared anthocyanins, flavonols, phenolic acids and derivatives, and VOCs between two typical fennel varieties with the application of ultra-high performance liquid chromatography coupled with quadrupole Orbitrap high-resolution mass spectrometry (UHPLC-Q-Orbitrap HRMS) and gas chromatography coupled to mass spectrometry (GC-MS). Furthermore, the critical transcription factor encoding genes such as AfTT8, AfMYB7, and AfTTG1 were carefully studied in tobacco and transgenic tomatoes, and it was proved that the transcriptional activation of AfTT8 is mainly responsible for the anthocyanin coloration and concomitant aroma change in purple fennel. Additionally, anthocyanin accumulation and aroma formation were systematically revealed for the first time by integrated metabolomic and transcriptomic analyses in aromatic herbs.

## Materials and Methods

### Materials

Fennel (*Anethum foeniculum* L.) seeds were obtained from Baker Creek Heirloom Seed Company. All the fennel plants including the green variety (Florence) and purple variety (Bronze) were grown in a greenhouse with a 16-h photoperiod at 25°C. Besides, tomato (*Solanum lycopersicon* Mill.) and tobacco plants (*Nicotiana benthamiana*) were also grown in the same conditions. The edible tender leaves were carefully sampled at 60 days after sowing seeds. Anthocyanin standards including pelargonidin-3-O-glucoside and cyanidin-3-O-glucoside and other phenolic compounds (quercetin-3-O-rutinoside, kaempferol-3-O-glucoside and chlorogenic acid) were purchased from PhytoLab (Germany). The other solvents and reagents were purchased from MERCK (Germany) and Sigma-Aldrich (St. Louis, MO).

### Identification and Quantification of Anthocyanins and Other Phenolic Compounds by UHPLC-Q-Orbitrap HRMS

Anthocyanins and the other phenolic compounds were extracted from tender fennel leaves with the method mentioned previously with minor modifications (21). Lyophilized sample (50 mg) was mixed with 1 ml mixture of 85% MeOH, 15% water, and 0.5% acetic acid in a tube. The suspension was vigorously vortexed for 15 min, sonicated for 15 min, and then placed on ice overnight. Next, the suspension was centrifuged at 13,000 g for 10 min, and the supernatant was filtered through a 0.22-μm PTFE membrane. The extraction for each sample was carried out for two times and the supernatant was mixed. The extract was analyzed by UHPLC-Q-Orbitrap HRMS (Thermo Scientific, USA) with the methods reported in the early study (22). It is worth mentioning that the ultra-high performance liquid chromatography was simultaneously coupled with a diode array detector (DAD) detector and a quadrupole Orbitrap high-resolution mass spectrometer in our laboratory. The UV–vis spectra and mass spectrometry of the individual anthocyanin separated by liquid chromatography were detected by DAD detector and quadrupole Orbitrap high-resolution mass spectrometer concurrently. About 1 μl of the extract was injected into a Vanquish Flex UHPLC system (Thermo Scientific) with a Waters ACQUITY UPLC HSS T3 column (1.7 μm, 2.1 mm × 100 mm). The eluates were first analyzed with a DAD detector. Anthocyanins and phenolic compounds were detected at the wavelength of 530 and 330 nm, respectively. A binary mobile phase consisting of acetonitrile (solvent A) and water (containing 0.1% formic acid; solvent B) was applied. Gradient elution program was as follows: initial, 2% A; at 1 min, 2% A; at 2 min, 8% A; at 8 min, 15% A; at 16 min, 18% A; at 26 min, 95% A; at 28 min, 95% A; at 29 min, 95% A; at 31 min, 2% A. The flow rate was set at 0.30 ml min−1. The Q-Exactive mass spectrometer was applied for the identification of the separated metabolites, and Xcalibur 2.3 software was applied for data acquisition. The Q-Exactive mass spectrometer was performed as the parameters reported before (22). Metabolites were identified by comparing their retention time, UV–vis spectra, polarity, and fragment of mass spectra with those of the authentic compounds (23–26). Individual anthocyanin content was quantified with peak area and calculated as the equivalent of pelargonidin-3-O-glucoside and cyanidin-3-O-glucoside as reported previously (21, 22). Similarly, flavonol content was quantified with peak area and calculated as the equivalent of quercetin-3-O-rutinoside and kaempferol-3-O-glucoside. Other phenolic compounds such as chlorogenic acid derivatives were calculated as the equivalents of chlorogenic acid. All the contents were expressed as mg g−1 dry weight.

### VOC Sampling and GC-MS Analysis

Volatile emission collections were carried out with the SPME method previously described (11). The identification and quantification of VOCs were conducted by a Model 7890B GC and a 7000D mass spectrometer (Agilent), equipped with a DB-5MS capillary column (5% phenyl-polymethylsiloxane, 30 m x 0.25 mm x 1.0 μm). The GC-MS data were unit variance scaled, and then, principal component analysis (PCA) was conducted. The hierarchical cluster analysis (HCA) results were presented as heatmaps. Pearson correlation coefficients (PCCs) were calculated by R. Differentially accumulated metabolites (DAMs) were characterized with the criterions of VIP ≥1 and absolute Log2-fold change ≥1. The VIP values were obtained from OPLS-DA (Orthogonal Partial Least Squares Discrimination Analysis) results. The metabolites identified with NIST were further annotated by KEGG compound database and further mapped to KEGG pathway. Metabolite sets enrichment analysis (MSEA) was applied to analysis pathways containing DAMs, using hypergeometric test's *p*-values to determine the significance.

### RNA-seq and Data Processing

Total RNAs were extracted from tender leaves and tested by standard methods reported previously (27). Thence, the constructed RNA-seq libraries were sequenced by MetWare Biotechnology Co., Ltd. (Wuhan, China, https://www.metware.cn) on NovaSeq 6000. All the downstream analyses were performed on the basement of high-quality clean RNA-seq data. *De novo* assembly was performed using Trinity (http://trinityrnaseq.sourceforge.net/) with parameters set in default. BUSCO v3 was applied to evaluate the transcriptome completeness. Transcriptome assembly quality was assessed by Transrate v1.0.3. TGICL (http://www.tigr.org/tdb/tgi/software/) was applied to remove the redundancy in transcripts to obtain the unigenes. The unigenes were annotated using BLASTX program (E-value <1E−5) against non-redundant (nr protein sequences) in NCBI (http://www.ncbi.nlm.nih.gov/), Swissprot (http://www.expasy.ch/sprot/), Gene Ontology (GO), Kyoto Encyclopedia of Genes and Genomes (KEGG) pathway (http://www.genome.jp/kegg/), Clusters of Orthologous Groups of Proteins (KOG/COG), and Pfam (https://pfam.xfam.org/). Differential expression analysis was conducted as the method reported previously (27).

### Bioinformatic Analysis

Multiple sequence alignment of the protein sequences was applied to DNAMAN software (version 6.0). Phylogenetic and molecular evolutionary analysis was performed with MEGA X.

### RNA Isolation and Quantitative Real-Time PCR Analysis

Total RNA extraction and RT-qPCR were performed with the protocols reported previously, and gene expression levels in fennel, tobacco, and tomato were normalized with β-actin, NbActin, and SlCAC (28). Each value represents three biological repeats.

### Plasmid Construction and Tobacco Transformation

The full-length open reading frame of AfMYB7 was amplified using primers AfMYB7OF (5′-GCTGTCTAGAACTATATCTTATTTACAGTGCTCA-3′) and AfMYB7OR (5′-TAGTGAGCTCGCTTACTAATTCACGGGTTA-3′). The amplification products were digested and inserted into plasmid pBI121 to create an overexpression vector Pro35S:AfMYB7. Similarly, vector Pro35S:AfTT8 was built with primers AfTT8OF (5′-GCTGTCTAGATCATAGCCAAGCTTGTGC-3′) and AfTT8OR (5′-TAGTGAGCTCTCCTCCATGTTCTGCTAATC-3′). All the expression vectors were further verified by sequencing and transformed into the leaves of Nicotiana benthamiana by agrobacterium-mediated transformation. Final measurements of anthocyanins in infiltrated tobacco leaves were taken at 4 days post-infiltration as described above (28).

### Co-Expression Vector Construction and Tomato Transformation

To build a co-expression vector for AfMYB7 and AfTT8, a fragment containing the full length of AfMYB7, a constitutive promoter 35SCaMV and a terminator Nos were amplified with primers ORFF (5′-CGGAATTCGCAGGTCCCCAGATTAGC-3′) and ORFR (5′-ACGCCAGGGTTTTCCCAGTCACGA-3′), using Pro35S:AfMYB7 plasmid as the template. Then, the amplified product was digested with EcoRI and cloned into Pro35S:AfTT8 vector, yielding a co-expression vector Pro35S:AfMYB7-Pro35S:AfTT8. The co-expression vector was finally verified by sequencing and transformed into tomatoes with the procedures reported before (28). The transgenic tomato lines were selected on kanamycin resistance and sequencing.

### Yeast Two Hybrid Assay

The carboxyl terminal-deleted form of AfTT8 was excised by EcoRI/SalI and linked to pGBKT7 to produce an in-frame fusion with the DNA binding domain of GAL4. Full-length sequences of AfMYB7, AfTTG1, and SlAN11 were digested with EcoRI/XhoI and linked to pGADT7 to produce an in-frame fusion with the activation domain of GAL4, respectively. The Y2H assay was carried out following the manufacturer's instructions (Clontech, CA, USA).

### Statistical Analysis of Data

Statistical analyses were conducted with SPSS 21.0, and the significance level was set at *p* < 0.05.

## Results and Discussion

### Anthocyanins Identified and Quantified in Different Fennel Varieties

**Figure 1:**
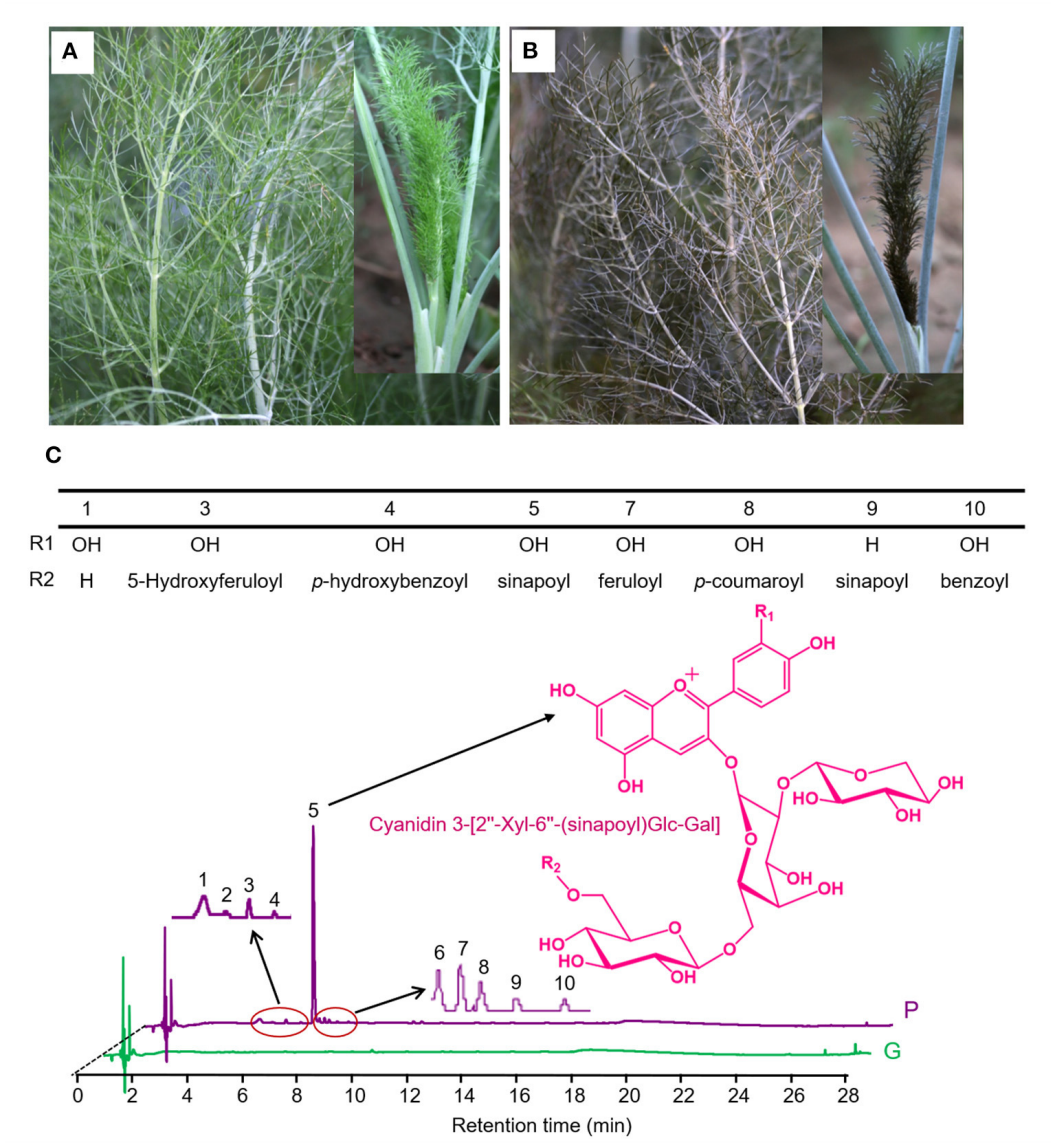
The structures of anthocyanins identified in purple fennel. **(A)** Phenotypes of the purple variety (Bronze, P) and **(B)** green variety (Florence, G). **(C)** The major anthocyanins in purple fennel were depicted as component 1 to 10 in chromatogram. Anthocyanins display a structural homology. The key differentiation is acyl groups (benzoyl, 4-hydroxybenzoyl, p-coumaroyl, caffeoyl, feruloyl, 5-Hydroxyferuloyl, and sinapoyl).

Visual inspection shows that “Bronze” displays intense accumulation of purple pigments in epidermal tissues of the whole fennel plant, compared with the green variety “Florence” (Figures 1A,B). Using UHPLC-Q-Orbitrap HRMS, these potential anthocyanins were identified and subsequently quantified in tender leaves of “Bronze” and “Florence.” A number of ten major peaks exhibiting typical light absorption at wavelength 530 nm were marked in the chromatogram and finally identified by comparing their UV-vis spectra, elution profile, and precursor and product ions to the literatures reported (23, 24) (Figure 1C and Table 1). The total anthocyanin content was found 18.17 mg/g (dry weight) of the edible tender leaves of “Bronze,” whereas none of the significant anthocyanins were found in the pigment extracts of the green variety “Florence” (Figure 1C and Table 1).

**Table 1:** Anthocyanin levels (mg/g dry weight) in leaves of the different fennel varieties (*n* = 3).

**No.^**a**^**	**RT^**b**^**	**λmax**	**[M+H]^**+**^ (m/z)**	**MS/MS** **(m/z)**	**Compound**	**calculated m/z**	**Error**	**Varieties**	
	**(min)**	**(nm)**				**(formula)**	**(ppm)**	***P***	**G**
1	4.31	514	743.2119	287.06	Cyanidin 3-(2”-Xyl-6”-Glc-Gal)	743.2029/C_43_H_49_O_24_	12	0.89 ± 0.01	^c^Nd
2	4.64	510	581.1581	287.06	Cyanidin 3-(2”-Xyl-Gal)	581.1501/C_26_H_29_O_15_	13	0.04 ± 0.01	Nd
3^d^	5.26	526	935.2578	287.06	Cyanidin 3-[2”-Xyl-6”-(5-Hydroxyferuloyl)Glc-Gal]	935.2451/C_42_H_47_O_24_	13	0.28 ± 0.02	Nd
4	5.75	526	863.2354	287.06	Cyanidin 3-[2”-Xyl-6”-(4-hydroxybenzoyl)Glc-Gal]	863.2240/C_39_H_43_O_22_	13	0.09 ± 0.01	Nd
5	6.17	530	949.2726	287.06	Cyanidin 3-[2”-Xyl-6”-(sinapoyl)Glc-Gal]	949.2608/C_43_H_49_O_24_	12	15.71 ± 0.04	Nd
6^d^	6.40	530	1111.3274	287.06	Cyanidin 3-[2”-(caffeoyl)Xyl-6”-(sinapoyl)Glc-Gal]	1111.2925/ C_52_H_55_O_27_	31	0.39 ± 0.03	Nd
7	6.60	526	919.2610	287.06	Cyanidin 3-[2”-Xyl-6”-(feruloyl)Glc-Gal]	919.2502/C_42_H_47_O_23_	12	0.29 ± 0.02	Nd
8	6.76	526	889.2501	287.06	Cyanidin 3-[2”-Xyl-6”-(p-coumaroyl)Glc-Gal]	889.2397/C_41_H_45_O_22_	12	0.23 ± 0.02	Nd
9^d^	7.05	518	933.2778	271.06	Pelargonidin 3-[2”-Xyl-6”-(sinapoyl)Glc-Gal]	933.2659/C_43_H_49_O_23_	13	0.15 ± 0.03	Nd
10^d^	7.45	530	817.2292	287.06	Cyanidin 3-[2”-Xyl-6”-(benzoyl)Xyl-Gal]	817.2185/C_38_H_41_O_20_	13	0.11 ± 0.01	Nd
Total								18.17 ± 0.08	Nd

a *No corresponds to the chromatographic peaks shown in Figure 1*.

b *RT, retention time*.

c *Nd, not detected. G, Florence. P, Bronze*.

d *Anthocyanins identified in fennel for the first time*.

*Xyl, Glc, and Gal indicate xylose, glucose, and galactose, respectively*.

Of the total anthocyanins, cyanidin-based anthocyanins account for most of the proportion (99%), while a thimbleful of pelargonidin-based anthocyanins was detected, indicating a very high hydroxylation efficiency at C3′ position of intermediate dihydrokaempferol in anthocyanin biosynthetic pathway. With respect to anthocyanin modification, the contents of acylated cyanidin glycosides (17.09 mg/g) occupied the absolute percentage (94%) of the total anthocyanins, and only a small amount of cyanidin glycosides were not acylated (5%). As the results shown in Table 1, nearly all the anthocyanidins (99%) undergone the same pattern of glycosylation modification at C3 position of the C ring. Subsequently, most of these glycosylated anthocyanins are further acylated by hydroxycinnamic acids which contain aromatic ring groups, unlike the anthocyanins widely found in vegetables and fruits such as blueberry, apple, okra, and other species (22, 24–27, 29). As acylated anthocyanins show significantly higher color stability than the un-acylated, the purple fennel might be good resource for natural food colorants (30).

It is worth noting that an acylated cyanidin glycoside, cyanidin 3-[2”-Xyl-6”-(sinapoyl)Glc-Gal], occupies a very large proportion (86%) of the total anthocyanins and is mainly responsible for the coloration of purple fennel. Taking these results into account, it can be concluded that glycosylated with trisaccharide at C3 position of anthocyanidin aglycones is a dominated modification pattern, and most of the glycosylated cyanidin were further acylated mainly by hydroxycinnamic acids, especially sinapic acid.

### Identification and Quantification of Other Phenolic Compounds

Similar to anthocyanins, other subgroups of phenolic compounds such as flavonols function as the important bioactive antioxidants by eliminating reactive oxygen species (ROS) which mainly result from the responses to UV irradiance, pathogen infection, and other stresses in plant and mammal cells (31–34). These phenolic compounds can also stabilize and contribute to the color formation of anthocyanins in beverage and foods through co-pigmentation (35, 36). As phenolic compounds show a typical UV-vis spectra absorption at 280–360-nm range, the 330-nm wavelength was selected for UHPLC analysis and thirteen candidate peaks were numbered in the chromatogram (Supplementary Figure S1). According to the data of UV–vis spectra, elution profile, and precursor and product ions provided by UHPLC-Q-Orbitrap HRMS, thirteen phenolic compounds were preliminarily identified (Table 2 and Supplementary Figure S1).

**Table 2:** Phenolic compounds (mg/g dry weight) in leaves of the different fennel varieties (*n* = 3).

**No.^**a**^**	**RT^**b**^**	**λmax; band I**,	**[M+H]^**+**^ (m/z)**	**MS/MS** **(m/z)**	**Compound**	**Calculated m/z**	**Error**	**Varieties**	
	**(min)**	**Band II (nm)**				**(Formula)**	**(ppm)**	***P***	**G**
1	4.71	242, 326	355.1068	163.04	Chlorogenic acid	355.1029/C_16_H_19_O_9_	10	2.24 ± 0.02^*^	1.24 ± 0.03
2	5.13	234, 294	355.1068	163.04	Cryptochlorogenic acid	355.1029/C_16_H_19_O_9_	10	0.05 ± 0.01	0.04 ± 0.01
3	9.05	246, 326	611.1682	449.11/287.06	kaempferol 3,4'-*O*-diglucoside	611.1612/C_27_H_31_O_16_	11	0.05 ± 0.01	0.03 ± 0.01
4	9.28	254, 354	611.1689	465.11/303.05	Quercetin 3-*O*-glucoside-7-*O*-rhamnoside	611.1612/C_27_H_31_O_16_	11	0.38 ± 0.02^*^	0.09 ± 0.01
5	9.62	254, 354	611.1689	465.11/303.05	Quercetin 3-*O*-rutinoside	611.1612/C_27_H_31_O_16_	11	0.69 ± 0.05	0.62 ± 0.03
6^d^	9.83	254, 354	479.1959	303.05	Quercetin-3-*O*-ferulic acid	479.0978/C_25_H_19_O_10_	205	2.56 ± 0.19	2.20 ± 0.18
7	10.00	254, 354	465.1092	303.05	Quercetin 3-*O*-glucoside	465.1033/C_21_H_21_O_12_	12	0.16 ± 0.02	0.20 ± 0.03
8	11.08	254, 354	435.0984	303.05	Quercetin 3-*O*-xyloside	435.0927/C_20_H_19_O_11_	13	0.29 ± 0.02^*^	0.61 ± 0.01
9^d^	11.27	250, 330	463.0932	287.06	Kaempferol 7-*O*-ferulic acid	463.1029/C_25_H_19_O_9_	20	0.12 ± 0.01^*^	0.02 ± 0.01
10^d^	12.14	246, 326	463.0932	287.06	Kaempferol 3-*O*-ferulic acid	463.1029/C_25_H_19_O_9_	20	1.80 ± 0.06^*^	0.72 ± 0.03

a *No corresponds to the chromatographic peaks shown in Supplementary Figure S1*.

b *RT, retention time*.

*^c^Nd, not detected. G, Florence. P, Bronze*.

d *Compounds identified in fennel for the first time*.

* *Represents significance (p < 0.05)*.

Of the total phenolic compounds in purple fennel, hydroxycinnamic acid derivatives and flavonol derivatives account for about 56 and 44%, respectively (Table 2). Apparently, the contents of most of the hydroxycinnamic acid derivatives including chlorogenic acid and cryptochlorogenic acid were enhanced evidently in purple fennel leaves (Table 2). Compared with the green variety, quercetin 3-*O*-glucoside-7-*O*-rhamnoside, and kaempferol 7-*O*-ferulic acid show significant increase, while quercetin 3-O-xyloside shows notable decrease in purple fennel leaf. Overall, the total flavonol content in purple fennel was significantly higher than the anthocyanin-less variety. Regardless of fennel variety, the chlorogenic acid occupies the highest proportion of the total hydroxycinnamic acid derivatives (Table 2). Among the total flavonol derivatives, quercetin-3-*O*-ferulic acid accounts for the highest proportion, following by kaempferol 3-*O*-ferulic acid which is remarkably different from the flavonol glycosides identified in other species such as okra, cowpea, and eggplant (22, 24, 26, 27, 29). Therefore, several conclusions on phenylpropanoid metabolism can be made initiatively: (1) the biosynthesis of caffeic acid and sinapic acid occupies an essentially important metabolic efflux in phenylpropanoid pathway in both fennel varieties; (2) flavonol aglycones in fennel are mainly decorated by ferulic acid other than saccharide; (3) quercetin-based flavonol derivatives are preferentially produced in flavonoid pathway in fennel. Collectively, it is evident that the profound accumulation of anthocyanins and other phenolic compounds (such as flavonols) are responsible for the coloration of purple fennel leaf, and “Bronze” is a good source of phenolic compounds for healthy human diet.

### Profound Effects on Aroma Formation Shed by Anthocyanin Accumulation

**Figure 2:**
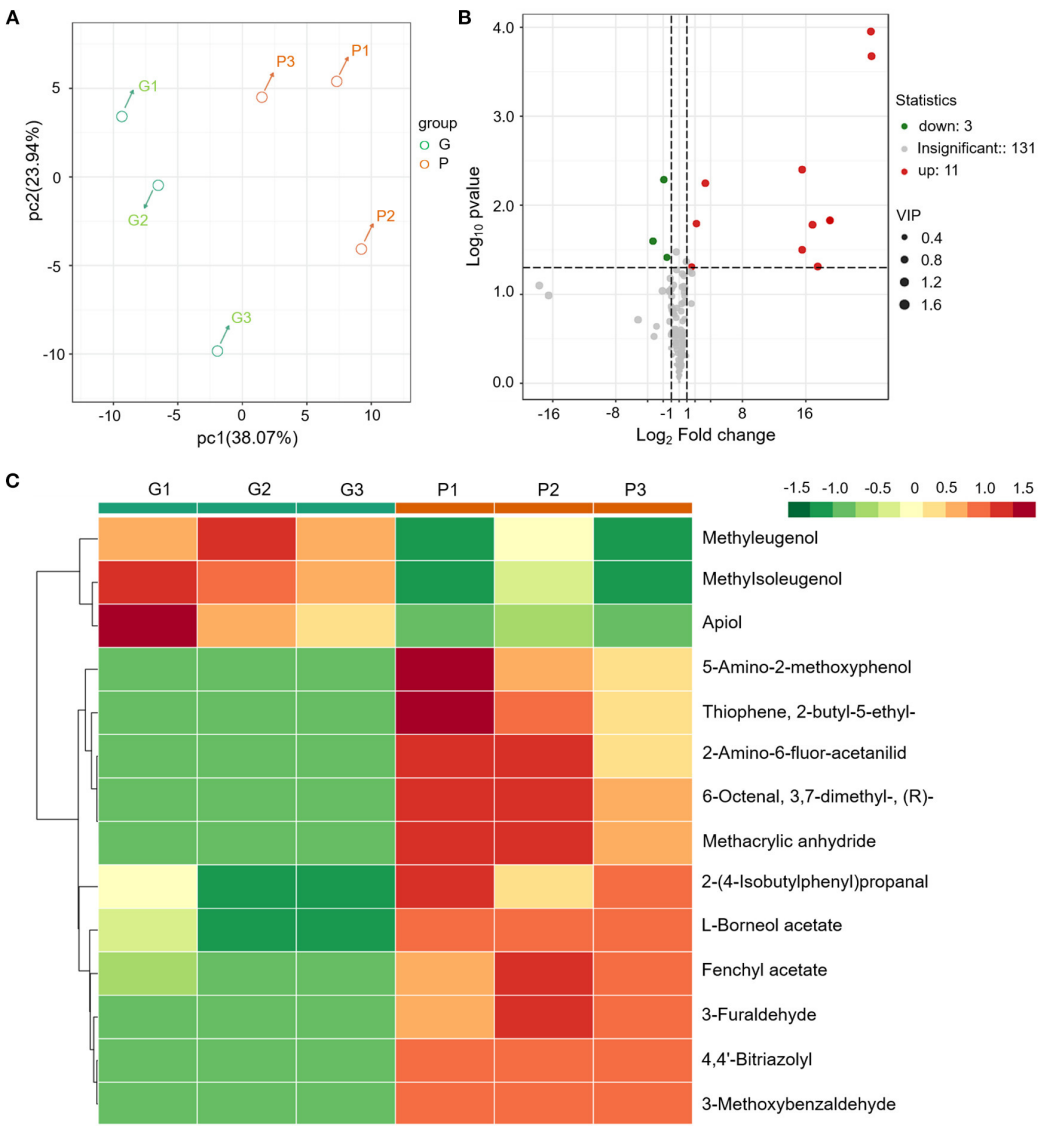
Metabolomics analysis of the VOCs reveals the aroma changes in different fennel varieties (G vs. P). **(A)** PCA of the different samples. **(B)** Different VOCs were indicated by volcano plots. **(C)** Cluster heat map of the DAMs of different varieties.

Compared with “Florence,” the aroma intensity of “Bronze” leaf has an evident decline in sensory. To study the relationship between the anthocyanin accumulation and aroma formation, a target metabolomic analysis of VOCs was performed. A total of 145 VOCs including terpenoids, benzenoids, esters, alcohols/aldehydes, phenylpropanoids, and unclassified compounds were detected (Figure 2 and Supplementary Table S1). PCA of the profiling data of these 145 VOCs showed the leaf tissues of the same variety grouped together and different varieties had distinct VOC profiles, indicating the SPME-GCMS method has a good reproducibility (Figures 2A,B).

In the purple fennel, a total of 14 differential accumulated metabolites (DAMs) were screened out (Figure 2B and Table 3). In detail, 14 DAMs include 3 volatile phenylpropanoids, 4 aldehydes, 2 esters, and 5 unclassified compounds. Clustering analysis showed that all the volatile phenylpropanoids (apiol, methyleugenol and methylisoeugenol) exhibited a significantly reduction in purple fennel (Figure 2C). On the contrary, all the other 11 DAMs that include aldehydes, esters, and unclassified compounds displayed an evident increase. Although some fennel-specific VOCs such as anethole and monoterpenes displayed no apparent changes, the fact that all the three volatile phenylpropanoids with characteristic aroma show evident reduction accompanied with a drastic enhancement of anthocyanins was reminiscent of a redistributed metabolic flux of phenylpropanoid pathway in purple fennel (17, 37). Collectively, the tremendous differences in levels of anthocyanins and volatile phenylpropanoids clearly explain the characteristic color and aroma formation of purple fennel. Additionally, the rich and diverse VOCs identified build a good foundation to identify novel metabolites related to aroma quality for the industry utilization and breeding of fennel.

**Table 3:** Differentially accumulated volatile metabolites in leaves of the different fennel varieties analyzed by GC-MS (*n* = 3).

**Compounds**	**Class**	**CAS**	**Match**	**RI**	**Nist RI**	**Relative quantification** ^**a**^		**Log_**2**_ FC**	***P* value**	**VIP**
		**Number**	**Factor**			**G**	***P***			
3-Methoxybenzaldehyde	Aldehydes	591-31-1	95.5	1,273.0	1,196	1	2.11 × 10^7^	24.33	2.12 × 10^−4^	1.68
4,4'-Bitriazolyl	-	16227-15-9	68.1	1,266.5	1,286	1	1.99 × 10^7^	24.25	1.12 × 10^−4^	1.68
6-Octenal, 3,7-dimethyl-, (R)-	Aldehydes	2385-77-5	95.2	1151.1	1152	1	5.51 × 10^5^	19.07	1.48 × 10^−2^	1.68
Methacrylic anhydride	-	760-93-0	62.2	1,151.1	1,054	1	5.51 × 10^5^	19.07	1.48 × 10^−2^	1.68
5-Amino-2-methoxyphenol	Phenols	1687-53-2	64.4	1,249.3	1,402	1	1.89 × 10^5^	17.52	4.88 × 10^−2^	1.68
2-Amino-6-fluor-acetanilid	-	18645-85-7	68.8	1,322.8	1,580	1	1.19 × 10^5^	16.87	1.66 × 10^−2^	1.68
3-Furaldehyde	Aldehydes	498-60-2	73.4	1,074.8	832	1	4.80 × 10^4^	15.55	3.98 × 10^−3^	1.68
Thiophene, 2-butyl-5-ethyl-	-	54411-06-2	62.2	1,315.8	1,223	1	4.81 × 10^4^	15.56	3.17 × 10^−2^	1.68
Fenchyl acetate	Monoterpenoids	13851-11-1	96.7	1,218.2	1,223	2.64 × 10^5^	2.63 × 10^6^	3.31	5.65 × 10^−3^	1.59
L-Borneol acetate	Monoterpenoids	5655-61-8	65.4	1,285.6	1,284	1.15 × 10^6^	5.25 × 10^6^	2.19	1.61 × 10^−2^	1.51
2-(4-Isobutylphenyl)propanal	Aldehydes	51407-46-6	67.4	1,647.8	1,464	8.35 × 10^5^	2.51 × 10^6^	1.58	4.91 × 10^−2^	1.32
Methyleugenol	Phenylpropanoids	93-15-2	84.7	1,398.4	1,402	1.25 × 10^6^	4.28 × 10^5^	−1.55	3.86 × 10^−2^	1.34
Methyisoleugenol	Phenylpropanoids	93-16-3	95.6	1,493.0	1,492	2.57 × 10^7^	6.72 × 10^6^	−1.93	5.17 × 10^−3^	1.49
Apiol	Phenylpropanoids	523-80-8	79.6	1,616.8	1,682	3.00 × 10^6^	3.03 × 10^5^	−3.31	2.53 × 10^−2^	1.51

a *Relative quantification was calculated by the area of each individual peak. G, Florence. P, Bronze. - indicate components of other classes*.

### Transcriptome Analysis of the Characterized Color and Aroma in Fennel

**Figure 3:**
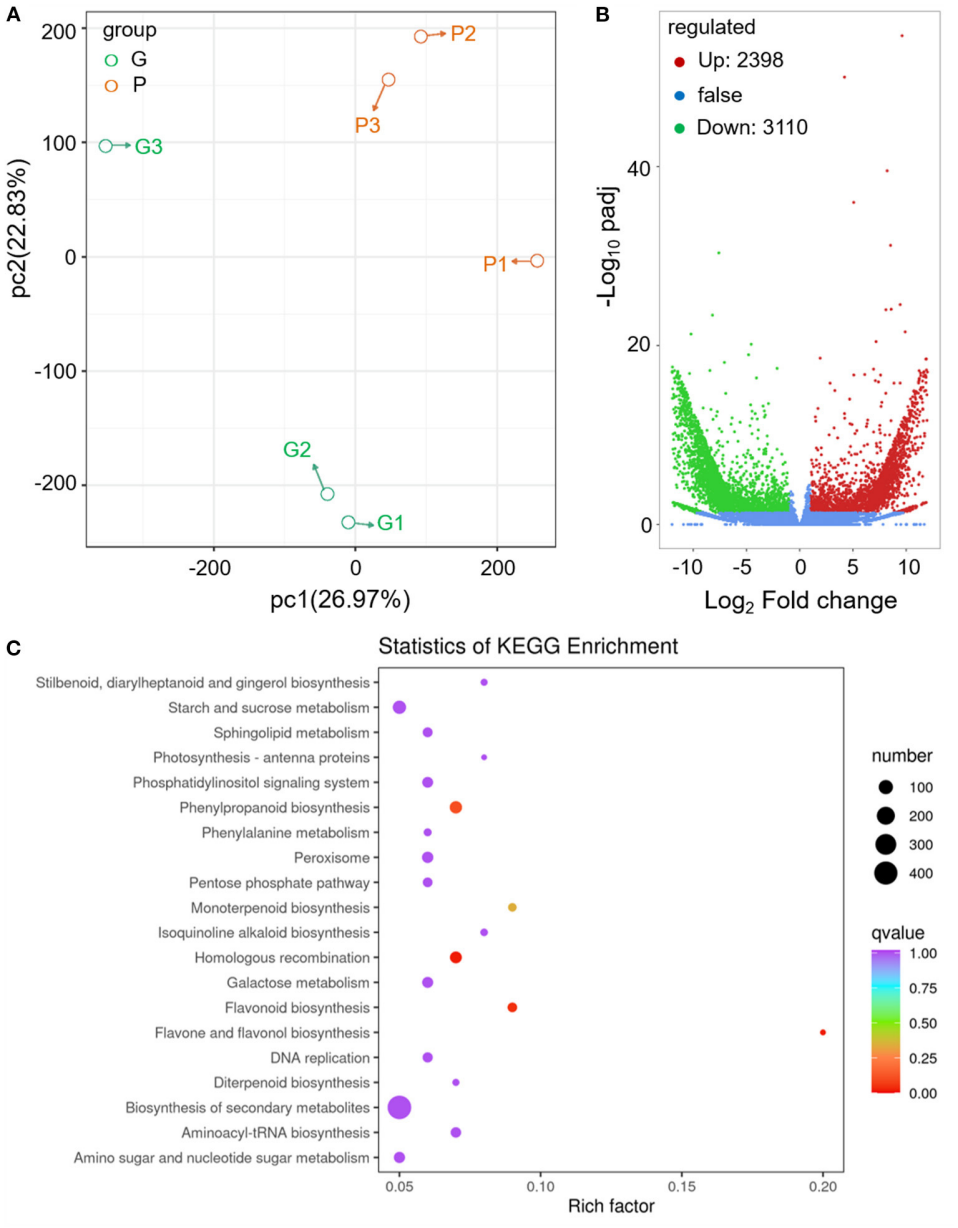
Transcriptomics analysis of differential gene expression between different fennel varieties. **(A)** PCA of the different fennel samples. **(B)** DEGs between different fennel varieties indicated with volcano plots (G vs. P). The horizontal axis represents the value of log2-fold change. **(C)** Scatter plots of KEGG pathway analysis.

To deeply understand the abundant accumulation of acylated anthocyanins and changed aroma in fennel, tender leaves of the two fennel varieties were sampled for transcriptome sequencing. The RNA-seq data are summarized in Supplementary Table S2. PCA and cluster analysis showed an acceptable reproducibility of the methods applied in transcriptome analysis (Figure 3A). Based on the results of transcriptome analysis, the transcripts of 15 selected differentially expressed genes (DEGs) were further analyzed by qRT-PCR (Supplementary Tables S3, S4), and an overall correlation coefficient indicates a good correlation between the data obtained by qRT-PCR and RNA-seq (Supplementary Figure S2).

Compared with the green variety, 2,398 genes were upregulated, and 3,110 genes were downregulated in purple fennel (Figure 3B). A number of 701, 125, and 112 unigenes were assigned to the biological process, molecular functional class, and cellular component, respectively, in the GO enrichment analysis of top 50 (Supplementary Figure S3). In “biological process” class, flavonoid biosynthetic process and anthocyanin-containing compound metabolic process rank first and fourth, respectively. In the “cellular component” category, most of the DEGs were annotated to involved in the assemblage of nuclear pore and SKI complex. Terpene synthase activity and amidase activity topped the rankings jointly in “molecular function” class. DEGs were also applied to KEGG analysis, and the results are displayed in Figure 3C. DEGs mapped to “biosynthesis of secondary metabolites” occupy the largest proportion, followed with “starch and sucrose metabolism” and “phenylpropanoid biosynthesis” pathway. In terms of rich factor, “flavone and flavonol biosynthesis,” “flavonoid biosynthesis,” and “monoterpenoid biosynthesis” rank in the top three pathway, indicating a similar finding in GO enrichment analysis that evident changes in gene expression levels associated with phenylpropanoid and terpenoid metabolism might be responsible for the diversities in leaf color and aroma between different fennel varieties (Figure 3 and Supplementary Figure S3).

### Redirected Metabolic Flux of Phenylpropanoid Pathway Accounts for the Intensive Anthocyanin Accumulation and Aroma Change in Purple Fennel

Although many DEGs subjected to monoterpenoid and diterpenoid metabolism were screened, all the terpenoids identified by GC-MS did not show evident differences in emission amounts (Supplementary Tables S1, S4). Gene families involved in terpenoid biosynthesis were identified in 16 plant species, and the copy number of these gene families varied greatly (38). Recently, phylogenetic analysis and Ks calculations indicate that large scale of duplications of these genes was almost all generated by the recent WGT event in *Tripterygium wilfordii* (38). Therefore, the inconsistency between transcriptome and metabolome results might arise from the high redundancy of terpene biosynthetic genes in fennel genome.

**Figure 4:**
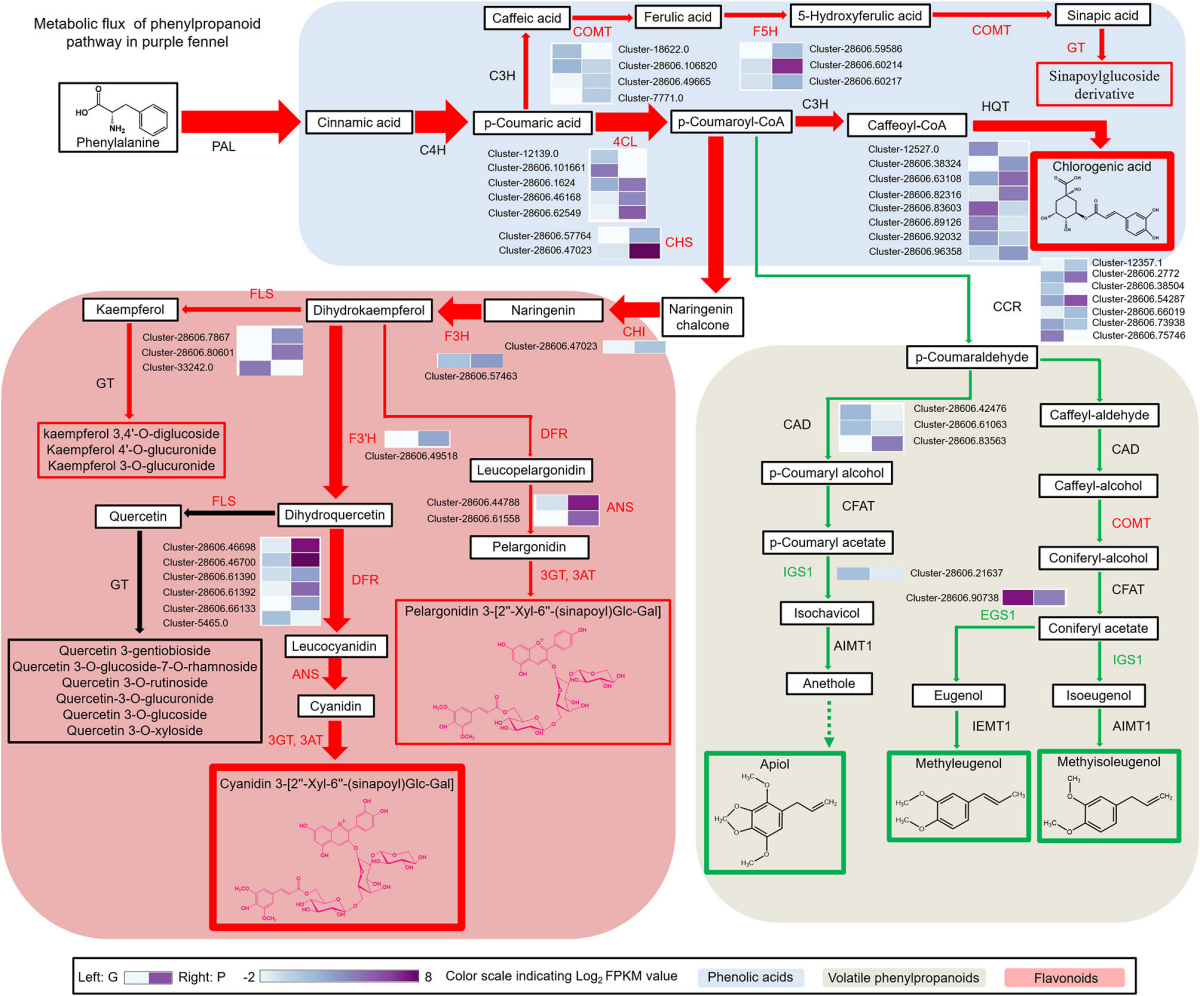
The metabolic flux redirection in phenylpropanoid pathway accounts for the characteristic aroma and color formation of the purple fennel. The red arrow indicates reinforced metabolic flux, while the green arrow indicates reduced metabolic flux. The width of the arrow indicates the corresponding biomass. The enhanced and repressed metabolites were marked with red and green frame, respectively. Transcript profiling of phenylpropanoid pathway genes was presented as grids with different colors.

Apparently, most of the structural genes of anthocyanin biosynthetic pathway were intensively upregulated in the purple leaves of “Bronze,” excepting a few very early biosynthetic genes (EBGs) of phenylpropanoid pathway including phenylalanine ammonia-lyase (PAL) and cinnamate 4-hydroxylase (C4H), in comparison with “Florence” (Figure 4 and Supplementary Table S4). Similar to the findings reported previously, the high gene transcripts (such as CHS, F3H, F3′H, DFR, and ANS) accord well with the drastically reinforced production of cyanidin-based anthocyanins in purple fennel (22, 28, 39). Similarly, the evidently upregulated transcripts of other structural genes such as *FLS* (flavonol synthase) might contribute to the enhanced accumulation of flavonols (Figure 4 and Supplementary Table S4). Particularly, it must be emphasized that the upregulated DFR and FLS function crucially in the allocation of metabolic flux toward anthocyanins and flavonols, because these two enzymes competitively bind dihydroflavonols as substrates (10, 40). Additionally, in the upstream phenylpropanoid pathway, the contents of most hydroxycinnamic acid derivatives showed evident increases in purple fennel, according well with the improved expression levels of the structural genes including 4CL (4-coumarateCoA ligase), COMT (caffeic acid 3-*O*-methyltransferase), and F5H (ferulate-5-hydroxylase) (Figure 4 and Supplementary Table S4). Unexpectedly, in terms of the remarkable decline of several volatile aroma phenylpropanoids such as methyleugenol, methyisoleugenol, and apiol in purple fennel, most of the structural genes exhibited stable expression levels between different varieties (Table 3, Figure 4, and Supplementary Table S4).

In anthocyanin biosynthetic pathway, it is apparent that the drastic upregulation of most structural genes results in abundant accumulation of acylated anthocyanins in purple fennel (Figure 4 and Supplementary Figure S2). In detail, ANS displayed the most significant upregulated scale about 2,000-folds in the comparison of G vs. P, followed by anthocyanidin glucosyltransferase (UGTs) and DFR which showed about 1,782-fold and 695-fold increase in purple fennel leaf, respectively (Supplementary Figure S2). Due to the drastically enhanced expression of F3′H, glycosyltransferases (GTs) and acyltransferase (SCPLs), a series of anthocyanins modified with hydroxycinnamic acids were generated (Figure 4 and Supplementary Figure S2). Combined with the results reported before, it is easy to conclude that the modulation of anthocyanin metabolism was dominated mainly by the simultaneous manipulation of a series of structural genes at mRNA level in fennel. Moreover, the upregulated structural genes should account for the enhanced production of flavonols and phenolic acid derivates through improving metabolic flux in respective biosynthetic pathways, based on the raised metabolic flux toward anthocyanins in purple fennel (Figure 4). In contrast to the highly reinforced accumulation of anthocyanins, flavonols, and phenolic acid derivates, all the volatile phenylpropanoids identified showed significant decline, indicating a repressed metabolic flux in biosynthetic pathway. Although most of the structural genes of volatile phenylpropanoids expressed stably between both fennel varieties, the drastically reinforced biosynthetic pathways for anthocyanins, flavonols, and hydroxycinnamic acid derivatives might probably achieve more metabolic flux through competitively binding substrates. In detail, serving as the substrates for chalcone synthase (CHS), cinnamate 3-hydroxylase (C3H), and cinnamoyl-CoA reductase (CCR), the allocation of p-coumaroyl-CoA in branch pathways of phenylpropanoid metabolism is the key factor which determines the metabolic flux and yield of the final products fundamentally (Figure 4). Distinctly, the substantially upregulated activities of C3H and CHS might allocate more substrates into the biosynthetic pathways of flavonoids and hydroxycinnamic acid derivatives in purple fennel, compared with the stable expression of CCR. Therefore, the decrease in volatile phenylpropanoids strongly indicates that the increased expression of structural genes of anthocyanins, flavonols, and hydroxycinnamic acid derivatives probably collaborates and redirects the metabolic flux along the phenylpropanoid pathway, as previously reported (40, 41).

### Identification of Critical Regulators Involved in Anthocyanin Regulation

**Figure 5:**
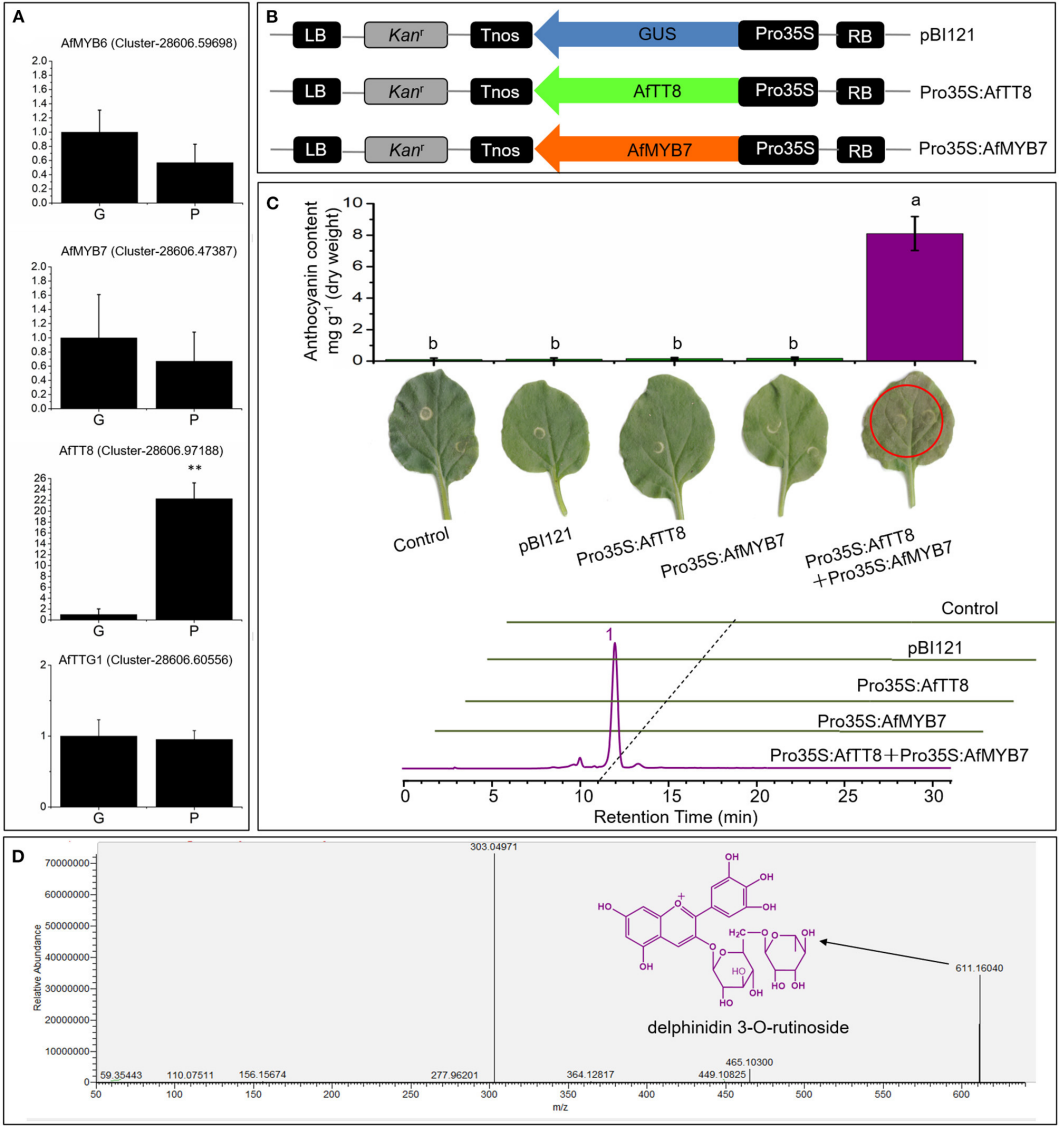
AfTT8 and R2R3-MYB protein AfMYB7 coordinately trigger anthocyanin accumulation in *Nicotiana benthamiana*. **(A)** Expression analysis of anthocyanin biosynthesis regulatory genes in leaves of different fennel varieties. **(B)** Schematic representation of the expression vectors. **(C)** Anthocyanin pigmentation and anthocyanin contents in *Nicotiana benthamiana* leaves (up) and HPLC profiles of anthocyanins extracted from infiltrated patches 4 days after infiltration (down). **(D)** MS^2^ spectra of the major anthocyanin [corresponding to peak 1 in the chromatogram of **(C)**] identified in infiltrated leaves and the structure was indicated with black arrow. Triplicate biological replicates were conducted and different letters represent significance at *p* < 0.05. G and P indicate the green and purple fennel variety, respectively.

An increasing number of evidences have revealed that EBGs including CHS, CHI, F3H, and F3′H are regulated by R2R3-MYB TFs such as MYB11, MYB12, and MYB111 in Arabidopsis, whereas the transcription of late biosynthetic genes (LBGs) including DFR, ANS, and UF3GT and other modification genes are directly regulated by the MYB-bHLH-WD40 (MBW) protein complex consisting of R2R3-MYB TFs, basic helix-loop-helix (bHLH) TFs, and WD40 repeat proteins, which have been found in many species (42). To reveal the molecular mechanisms of the characteristic color and aroma formation in purple fennel, a total of 273 DEGs encoding transcription factors were screened out and annotated to 16 gene families, such as MYB and bHLH (Supplementary Tables S5, S6). Expectedly, none of the identified WD40 repeat proteins show obvious expression changes between two fennel varieties, similar to the previous reports (Figure 5A and Supplementary Table S5) (22, 27, 28, 42). Compared with the green fennel, AfTT8, a bHLH transcription factor, exhibited significant upregulation in purple fennel leaves, indicating a critical function in the regulation of anthocyanin production. Additionally, other putative regulatory proteins of MYB family associated with anthocyanin biosynthesis showed similar expression levels between different fennel varieties.

### AfTT8 and AfMYB7 Coordinately Regulate Anthocyanin Biosynthesis in Tobacco

**Figure 6:**
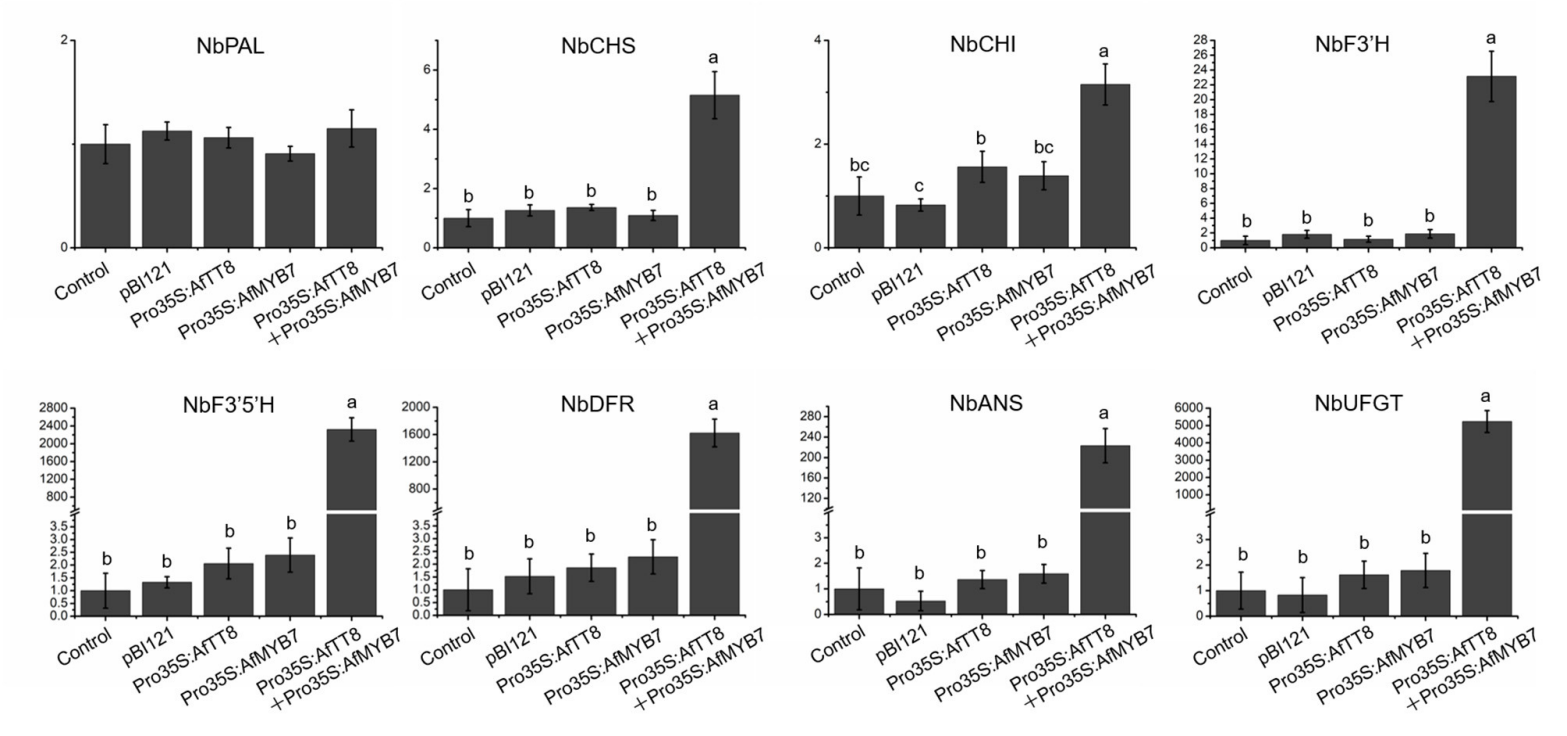
Expression analysis of anthocyanin biosynthetic genes in tobacco leaves infiltrated by *Agrobacterium tumefaciens* with different expression constructs. The longitudinal axis represents relative gene expression normalized to *NbActin*. Triplicate biological replicates were conducted and different letters represent significance at *p* < 0.05.

Bioinformatic analysis showed that AfTT8 contains a conserved basic bHLH domain and a MYB interaction region (Supplementary Figure S4). Further phylogenetic analysis indicates that AfTT8 is an ortholog of anthocyanin regulator, MdbHLH3 (Supplementary Figure S5). Therefore, expression vectors were built and applied for transient expression assays in tobacco (Figure 5B). Obviously, AfTT8 failed to trigger anthocyanin accumulation in tobacco leaves alone, indicating the insufficient gene expression levels of endogenous anthocyanin regulators of MYB family or low interaction efficiency between AfTT8 and other endogenous protein members of MBW complex in tobacco (28). Subsequently, the inability of AfMYB7, an ortholog of DcMYB7, to trigger anthocyanin biosynthesis alone was also found in infiltrated tobacco leaves, further indicating the important function of MBW complex. Moreover, it was demonstrated that R2R3-MYB protein DcMYB7 and DcMYB113 function essentially in regulating anthocyanin accumulation in carrot, indicating that MYB-bHLH interaction might be an essential prerequisite for anthocyanin regulation in Umbelliferae plants (40, 43). Therefore, a combination of AfMYB7 and AfTT8 expression vector was introduced into tobacco leaves, and intensive anthocyanin accumulation was induced (Figure 5C). Delphinidin 3-O-runtinoside was further identified to be the most important anthocyanin produced in infiltrated patches of tobacco leaves by UHPLC-Q-Orbitrap HRMS (Figure 5D), indicating a slightly different anthocyanidin modification pathway between tobacco and fennel. Moreover, the transcripts of most anthocyanin biosynthetic genes were examined in all the infiltrated patches of tobacco leaves, and the results showed that most of the structural genes were upregulated significantly under the coordinate regulation of AfTT8 and AfMYB7 (Figure 6). Altogether, it can be concluded that AfTT8 and AfMYB7 coordinately trigger anthocyanin accumulation in transiently transfected tobacco cells by activating the expression of biosynthetic genes. However, the functions of AfTT8 and AfMYB7 in stably transformed plants were still unknown.

### A Hybrid MBW Complex Triggers Anthocyanin Biosynthesis in Tomato

**Figure 7:**
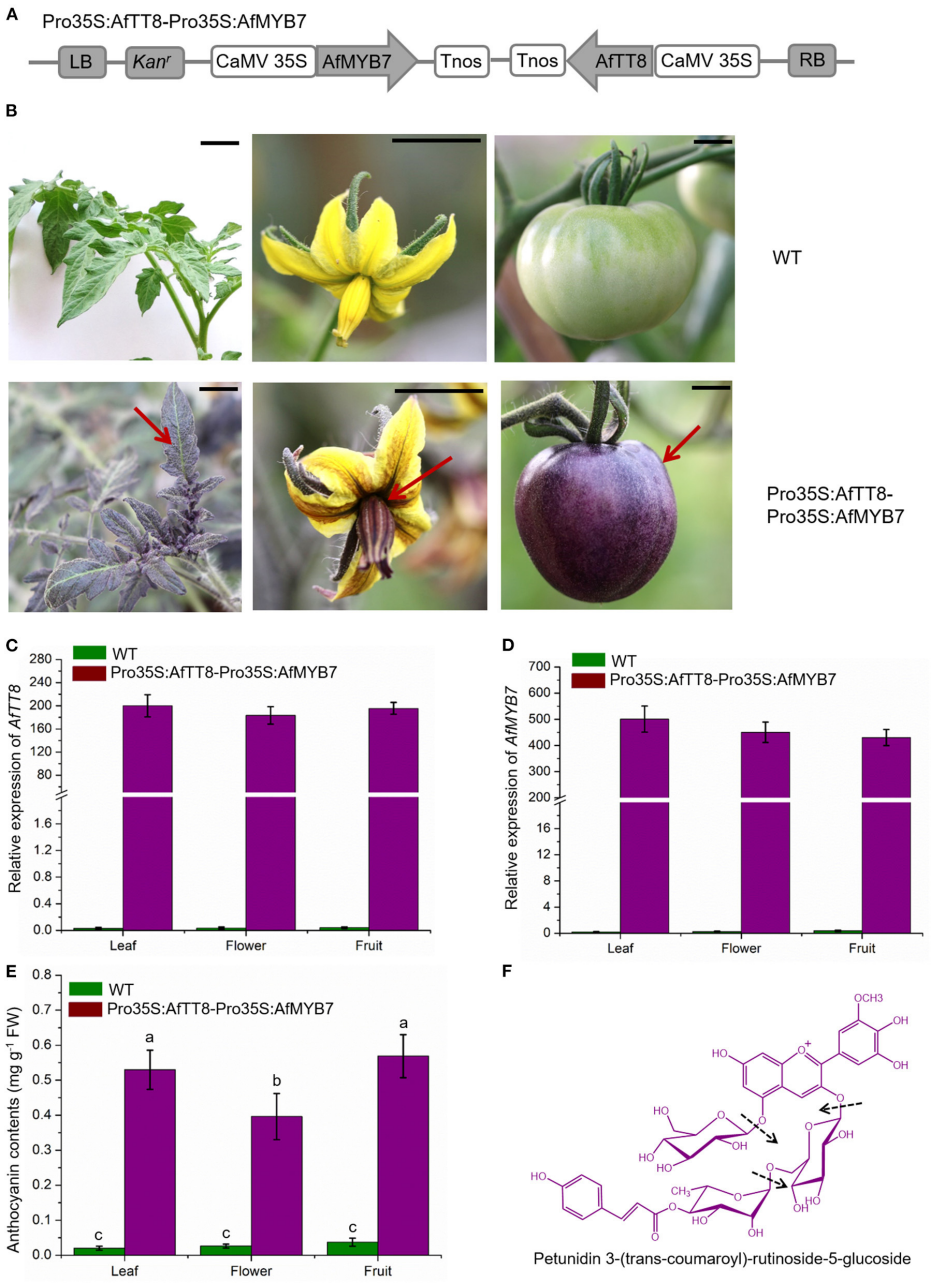
AfMYB7 and AfTT8 coordinately trigger anthocyanin biosynthesis in various tomato tissues. **(A)** Schematic representation of the co-expression vector Pro35S:AfTT8-Pro35S:AfMYB7. **(B)** Phenotypes of WT (wild type) and transgenic tomato plants. Expression analysis of *AfTT8* **(C)** and *AfMYB7* **(D)** in WT and transgenic tomato lines. Gene expression levels were normalized to *SlCAC*. **(E)** The contents of total anthocyanins in various tissues. **(F)** Structure of the major anthocyanin petunidin 3-(trans-coumaroyl)-rutinoside-5-glucoside in reference to peak 3 in Supplementary Figure S6A and the cleavage sites were annotated by arrows. Triplicate biological replicates were conducted and different letters represent significance at *p* < 0.05.

To further study the function of AfTT8 and AfMYB7 in regulating anthocyanin biosynthesis, a co-expression vector Pro35S:AfMYB7-Pro35S:AfTT8 was built as the schematic presentation shown in Figure 7A and introduced into wild type (WT) tomato by *Agrobacterium tumefaciens*-mediated transformation. Compared with the WT tomato plants, Pro35S:AfMYB7-Pro35S:AfTT8 plants (T0 generation) that showed high gene expression levels of *AfTT8* and *AfMYB7* all exhibited abundant accumulation of anthocyanins in the epidermal tissues of leaves, flowers, and fruits (Figure 7). Using UHPLC-Q-Orbitrap HRMS, the components of anthocyanins were identified and quantified, and petunidin 3-(trans-coumaroyl)-rutinoside-5-glucoside was found the most abundant components in fruits (Figures 7E,F, and Supplementary Figure S6). Apparently, total anthocyanin contents in leaves, flowers, and fruits of Pro35S:AfMYB7-Pro35S:AfTT8 plants were significantly higher than those in WT plants (Figure 7E). Further study indicates that the critical anthocyanin biosynthetic genes (*SlF3*′*5*′*H, SlDFR*, and *SlANS*) were drastically upregulated in the corresponding tissues of Pro35S:AfMYB7-Pro35S:AfTT8 plants, in comparison with WT (Supplementary Figure S7A). As F3′5′H (flavonoid 3′5′-hydroxylase) efficiently hydroxylated dihydroquercetin at position 3′ and 5′ to produce dihydromyricetin, the drastic upregulation of *SlF3*′*5*′*H, SlDFR*, and *SlANS* triggered by AfTT8 and AfMYB7 provided a great explanation for the abundant accumulation of delphinidin-based anthocyanins in transgenic tomato fruit. Altogether, these results clearly proved that AfTT8 and AfMYB7 intensively and widely trigger anthocyanin biosynthesis in transgenic tomato *via* activating the structural genes at mRNA level.

Previous studies have proven that WD40 proteins participate in kinds of life activities that include anthocyanin and seed coat mucilage biosynthesis, trichome development, and root hair patterning (44). Although WD40 repeat proteins usually expressed stably in different tissues or environmental conditions, TTG1 functions importantly in most MYB-bHLH interactions mainly by stabilizing the MBW complex (44). Multiple sequence alignment of anthocyanin regulators of bHLH family showed that AfTT8 contains a conserved bHLH domain and a Myb interaction region (Supplementary Figure S4). To investigate the mechanisms underlying the abundant anthocyanin accumulation in Pro35S:AfMYB7-Pro35S:AfTT8 plants, Y2H assays were conducted to analyze the physical interaction between AfTT8 and other candidate transcription factors. The results showed that AfTT8 efficiently interacted with AfMYB7 and SlAN11 (an orthologous protein of AtTTG1) directly in yeast (Supplementary Figure S7). Altogether, these results show that a hybrid MYB-bHLH-WD40 complex consisting of AfTT8, AfMYB7, and SlAN11 intensively and widely triggers anthocyanin accumulation in transgenic tomato. Similarly, these results suggest the hypothesis that a ternary MBW complex consisting of AfTT8, AfMYB7, and AfTTG1 might regulates anthocyanin biosynthesis fundamentally in fennel.

### A Ternary MBW Complex Consisting of AfTT8, AfMYB7, and AfTTG1 Mainly Regulates Anthocyanin Accumulation and Aroma Change in Purple Fennel

To further confirm the hypothesis that a ternary MBW complex fundamentally regulates anthocyanin accumulation in purple fennel, Y2H assays were conducted and it was proved that AfTT8 can interact with AfTTG1 (an orthologous protein of AtTTG1) physically (Supplementary Figure S7). Lots of reports have proven the LBGs essential for anthocyanin biosynthesis are directly regulated by MBW complex consisting of proteins of R2R3-MYB, bHLH, and WD40 family, although the proteins of each family might vary greatly in plants (28, 42). For instance, anthocyanin biosynthesis is directly triggered by the coordinate regulation of R2R3 MYB protein (MYB75, MYB90, MYB113, and MYB114), bHLH protein (GL3, EGL1/EGL3, and TT8), and WD40 protein (TTG1) in Arabidopsis. Although WD40 repeat proteins usually expressed stably in different tissues or environmental conditions, TTG1 functions importantly in MBW complex assemblage by stabilizing MYB-bHLH interaction (44). The results of Y2H, transient tobacco expression assays, and transgenic tomato studies show that WD40 repeat protein AfTTG1 could efficiently interact physically with AfTT8, further yielding the assemblage of a ternary MBW complex in purple fennel. In this study, a bHLH transcription factor encoding gene *AfTT8* that shows high gene expression in purple fennel leaves was isolated and studied in depth. Whereas, most potential anthocyanin regulators of MYB and WD40 family expressed stably between the two fennel varieties. Function studies show co-expression of AfTT8 and AfMYB7 significantly enhanced the metabolic flux toward anthocyanins in both tobacco and tomato by transcriptional activating most of the structural genes (Figures 5–7 and Supplementary Figure S7). Together with the results of Y2H assays, these results clearly prove that a ternary MBW complex consisting of AfTT8, AfMYB7, and AfTTG1 functions essentially in regulating anthocyanin accumulation in purple fennel.

**Figure 8:**
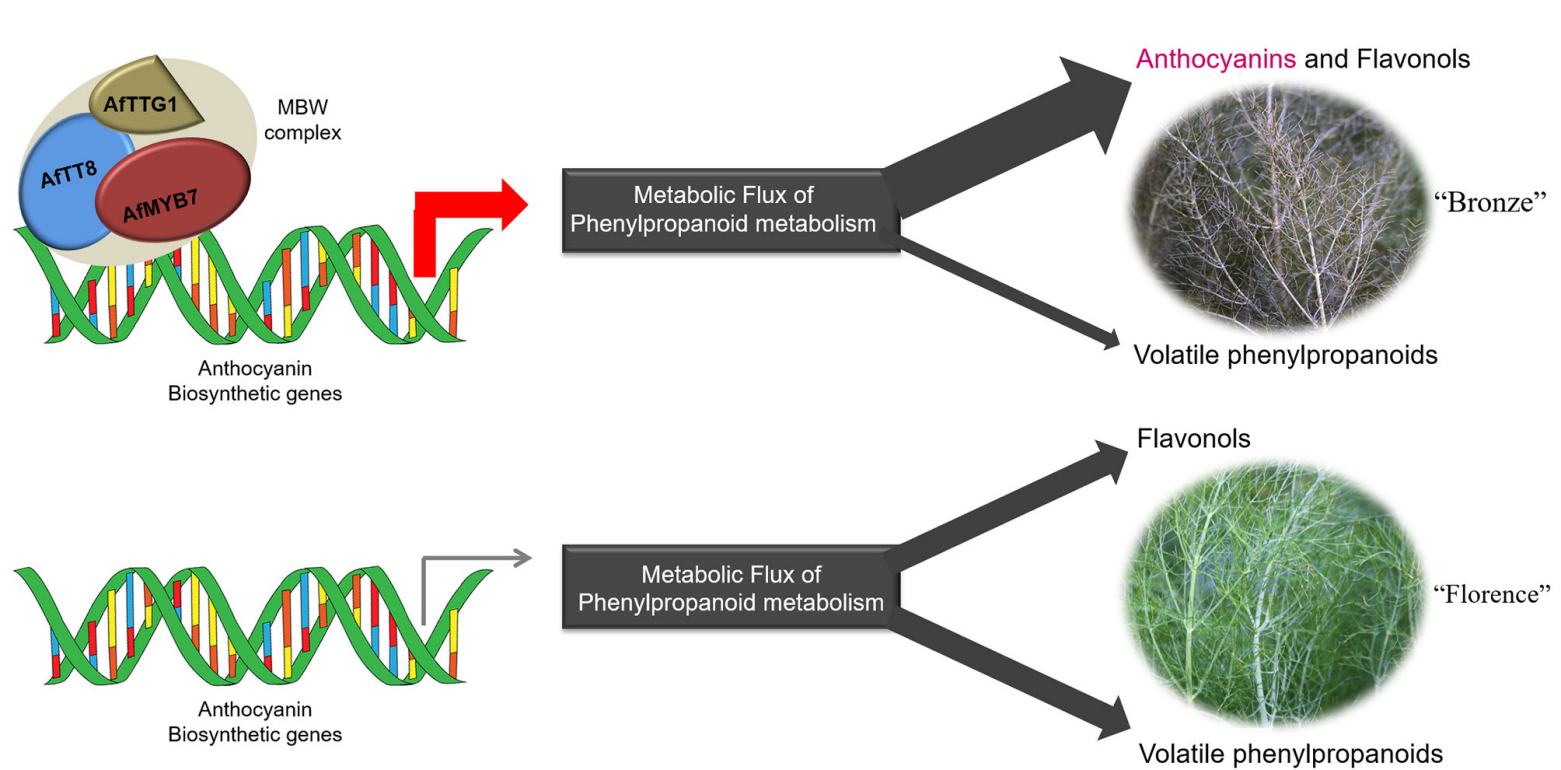
The ternary MBW complex consisting of AfTT8, AfMYB7 and AfTTG1 regulates anthocyanin coloration and aroma formation in fennel through activating the anthocyanin biosynthetic genes at mRNA level and redirecting the metabolic flux of phenylpropanoid metabolism.

In plants, bHLH proteins belong to the second largest transcription factor family (bHLH superfamily) and widely participate in the regulation of various biological processes such as response to environmental and hormone signals, secondary metabolisms, cell fate determination, and developmental patterns in root and flower (45). However, studies about the ortholog proteins of AfTT8 directly participate in the regulation of volatile phenylpropanoids have not yet been reported in plants (45). Furthermore, most of the structural genes responsible for the production of volatile phenylpropanoids show barely expression changes between different fennel varieties. Upon the studies on phenylpropanoid and terpenoid biosynthesis, the manipulations of pathway genes and their transcriptional regulators could exert significant effects on metabolic flux redirection in genetic modified plants (26, 46–49). Thus, it can be concluded that the upregulated structural genes triggered by the MBW complex consisting of AfTT8, AtMYB7, and AfTTG1 could greatly enhance the metabolic flux toward anthocyanins and collaborate with the upregulated C3H, F5H, COMT, and FLS in reprocessing phenylpropanoid pathway, resulting in the repressed metabolic flux into the branch pathway toward volatile phenylpropanoids in purple fennel. Therefore, the evident decline in volatile phenylpropanoids further led to the aroma intensity change in purple fennel leaf. Finally, a model summarizing the characteristic anthocyanin pigmentation and aroma formation in purple fennel was proposed (Figure 8). The transcriptional activation of a bHLH transcription factor encoding gene AfTT8 promotes the assembly of a ternary MBW complex consisting of AfTT8, AfMYB7, and AfTTG1, which intensively triggers anthocyanin biosynthesis through directly regulating the expression of structural genes at mRNA level. Subsequently, the upregulated expression of both anthocyanin structural genes and *FLS, C3H, F5H*, and *COMT* genes jointly enhanced metabolic flux toward anthocyanins, flavonols, and hydroxycinnamic acid derivatives and resulted in the evidently reduced production of the volatile phenylpropanoids in purple fennel leaf, in comparison with the green fennel leaf.

Excepting the anthocyanin activators of MYB family, some negative transcriptional regulators including the R3-MYB proteins (CPC and MYBL2) and R2R3-MYB protein (MYB4) that block anthocyanin biosynthesis *via* inhibiting functional MBW complex assembly or directly repressing the transcription of structural genes (28, 42, 50–53). However, most of these potential repressors show stable expression levels between the different fennel varieties (Supplementary Table S4). Although anthocyanin accumulation is mainly regulated by the MBW complex at mRNA level, transcriptional and post-translational modulations of the MBW complex widely participate in the fine-tune of anthocyanin biosynthesis in plants under ever-changing environments (42). For instance, ANL2, a homeodomain protein, was found to be necessary for anthocyanin accumulation in vegetative tissues (42). Moreover, some other differentially expressed transcription factors such as WRKY proteins suggest the mechanisms underlying the characteristic color and aroma formation in purple fennel might be more complicated than we proposed and more studies are required to clarify these problems (Supplementary Table S5).

## Conclusion

In summary, we identified the main anthocyanin pigments and VOCs responsible for the color and aroma discrepancies between different fennel varieties for the first time and found that the metabolic flux redirection of phenylpropanoid pathway primarily regulated by a functional MBW complex which consists of AfTT8, AfMYB7, and AfTTG1 mainly accounts for the characteristic anthocyanin coloration and aroma formation of purple fennel. Additionally, UHPLC-Q-Orbitrap HRMS analysis indicates that the purple fennel is a good source of phenolic compounds for healthy human diet and natural dye, and the rich and diverse VOCs identified build a good foundation to identify novel metabolites related to aroma quality for the industry utilization and breeding of fennel. Altogether, the systematic understanding of the anthocyanin accumulation and propound effects on aroma formation will assist in the further utilization and improvement of fennel resource.

## Data Availability Statement

The datasets presented in this study can be found in online repositories. The names of the repository/repositories and accession number(s) can be found below: https://www.ncbi.nlm.nih.gov/, PRJNA762379.

## Author Contributions

YZ performed the vector construction and generated the transgenic tomato plants. YF and QY performed the UHPLC-Q-Orbitrap HRMS assays. QZ performed the transient expression assays in tobacco, and TZ performed and analyzed the Y2H experiments. HG and YD conducted the analysis of omics data. YZ, JH, and YL wrote and reviewed the article, with assistance from all co-authors. All authors contributed to the article and approved the submitted version.

## Funding

This work was supported by the National Natural Science Foundation of China (Grant No. 31601760) and Henan Provincial Department of Science and Technology Research Project (182102110244).

## Conflict of Interest

The authors declare that the research was conducted in the absence of any commercial or financial relationships that could be construed as a potential conflict of interest.

## Publisher's Note

All claims expressed in this article are solely those of the authors and do not necessarily represent those of their affiliated organizations, or those of the publisher, the editors and the reviewers. Any product that may be evaluated in this article, or claim that may be made by its manufacturer, is not guaranteed or endorsed by the publisher.

## Supplementary Material

Supplementary Table S1 Identification and quantification of the VOCs in the leaves of different fennel varieties.

Supplementary Table S2 Primers used for gene expression analysis by RT-qPCR.

Supplementary Table S3 Statistical summary of the *de novo* assembly for the different samples.

Supplementary Table S4 All the DEGs between the different fennel varieties.

Supplementary Table S5 All the DEGs encoding transcription factors between the different fennel varieties.

Supplementary Table S6 A summary of the differentially expressed transcription factors in fennel leaves (G vs. P).
