# Supplementary material for: Integrated Transcriptomic and Metabolomic Analyses Reveal the Mechanisms Underlying Anthocyanin Coloration and Aroma Formation in Purple Fennel
Source: Front Nutr. 2022 Apr 27;9:875360. doi: 10.3389/fnut.2022.875360 (PMC9093692; doi:10.3389/fnut.2022.875360)
Supplement: Supplementary Table S2 — Primers used for gene expression analysis by RT-qPCR. [file Table_2.DOCX]

**Supplemental Table 2.** Statistical summary of the de novo assembly for the different samples.

| Sample | G1 | G2 | G3 | P1 | P2 | P3 |
| --- | --- | --- | --- | --- | --- | --- |
| Raw Reads | 433 741 34 | 418 594 72 | 435 688 18 | 434 799 64 | 443 860 28 | 429 014 88 |
| Clean Reads | 411 171 68 | 400 985 54 | 415 224 52 | 419 866 12 | 428 061 62 | 407 655 68 |
| Clean Base (G) | 6.17 | 6.01 | 6.23 | 6.3 | 6.42 | 6.11 |
| Error Rate (%) | 0.03 | 0.03 | 0.03 | 0.03 | 0.03 | 0.03 |
| CleanQ20 (%) | 97.68 | 97.63 | 97.57 | 97.53 | 98.12 | 97.49 |
| CleanQ30 (%) | 93.28 | 93.15 | 93.03 | 92.96 | 94.24 | 92.85 |
| GC Content (%) | 43.06 | 42.68 | 43.02 | 43.07 | 43.15 | 43.17 |
