# Supplementary material for: Integrated Transcriptomic and Metabolomic Analyses Reveal the Mechanisms Underlying Anthocyanin Coloration and Aroma Formation in Purple Fennel
Source: Front Nutr. 2022 Apr 27;9:875360. doi: 10.3389/fnut.2022.875360 (PMC9093692; doi:10.3389/fnut.2022.875360)
Supplement: Supplementary Table S3 — Statistical summary of the de novo assembly for the different samples. [file Table_3.DOCX]

**Supplemental Table 3.** Primers used for gene expression analysis by RT-qPCR.

| Genes | Primer sets | Accession |
| --- | --- | --- |
| *AfCHS* | F: 5'-AGCCCAAACCATTCTTCC-3'  R: 5'-ATGCCTCAACTAAACTCTTCCT-3' | Cluster-28606.57764 |
| *AfDFR* | F: 5'-TTGCATTTCTCGCTTATATACC-3'  R: 5'-AACATTGACAGTTCCGGC-3' | Cluster-28606.66133 |
| *AfANS* | F: 5'-CCCAAATGTCCTCAACCA-3'  R: 5'-AGCCCAAGAAATCCTAACC-3' | Cluster-28606.61558 |
| *AfUGT1* | F: AAGTGGAAGAAGGCAGCAG  R: CTATACTATAATAAATAATGTGTGCG | Cluster-28606.50632 |
| *AfUGT2* | F: ACAGCATAGACTGGAGGGA  R: GAAAAAGGATCTGAAGTAACG | Cluster-28606.88750 |
| *AfUGT3* | F: TTGTTGGCCTTTTTTTGCC  R: CGCTTTCTTCCTCATTTCGT | Cluster-25380.0 |
| *AfUGT4* | F: TGAATGCAACACTGCGAAC  R: CCAAGCGGTGAATAATAATA | Cluster-28606.54335 |
| *AfUGT5* | F: AAAAACAAGAACGCCAACA  R: TCTAACCCTAATCCACCCG | Cluster-28606.60134 |
| *AfUGT6* | F: GAGAAGAAGATGGGGCGAT  R: TGAAGTTGTGGATGTAAGAGGA | Cluster-16479.0 |
| *AfSCPL1* | F: TTCAGTTATAGCTCTGATAAGCG  R: ATGCAGGAATGTAGTGGCC | Cluster-28606.67072 |
| *AfSCPL2* | F：TTCCTTTGGTTGAACAGATG  R：TAAATACTTTAATGAATGGCGT | Cluster-28606.14802 |
| *AfSAT* | F: 5'-CTACAGGGGAATGGGAAA-3'  R: 5'-GGCTTGAGTTGATTGAAATG-3' | Cluster-28606.39897 |
| *AfBAHD1* | F: 5'-CCAGTCCCCTAAAATGTTCC-3'  R: 5'-AAATGTGTTATCCGTTCCGTA-3' | Cluster-28606.89867 |
| *AfBAHD2* | F: GCAACCCTATCATCTTCCA-3'  R: AACCACTCCTCACACTCTCC-3' | Cluster-28606.56732 |
| *AfBAHD3* | F: CTTTTCTCGTCTTCTTTCGC-3'  R: CCACCATTATTCCATCTCCA-3' | Cluster-28606.55483 |
| *AfMYB6* | F: 5'-GGATTGAGAAAGCACGGTA-3'  R: 5'-AAGAGGTGGTGAGAGGGGA-3' | Cluster-28606.59698 |
| *AfMYB7* | F: CGATTCAATCCTGCTCTCT-3'  R: CTAAACCTTCAACCCTGCG-3' | Cluster-28606.47387 |
| *AfTT8* | F: 5'-CTGAGGATGAGTTACAGGAACA-3'  R: 5'-GGGGGGAATGGATAGAATA-3' | Cluster-28606.97188 |
| *AfTTG1* | F: 5'-ATATCATTTTGTTTCCCCG-3'  R: 5'-GTCAATCTCCTCTTCCCCT-3' | Cluster-28606.60556 |
