# Supplementary material for: Integrated Transcriptomic and Metabolomic Analyses Reveal the Mechanisms Underlying Anthocyanin Coloration and Aroma Formation in Purple Fennel
Source: Front Nutr. 2022 Apr 27;9:875360. doi: 10.3389/fnut.2022.875360 (PMC9093692; doi:10.3389/fnut.2022.875360)
Supplement: Supplementary Table S6 — A summary of the differentially expressed transcription factors in fennel leaves (G vs. P). [file Table_6.DOCX]

**Supplemental Table 6.** A summary of the differentially expressed transcription factors in fennel leaves (G vs. P).

| Gene Family | Number of  DEGs | Upregulated  DEGs | Downregulated  DEGs | Description | Biological functions |
| --- | --- | --- | --- | --- | --- |
| MYB | 19 | 5 | 14 | MYB TFs | Development and phenylpropanoid pathway |
| bHLH | 22 | 11 | 11 | bHLH TFs | Development and secondary metabolism |
| bZIP | 6 | 2 | 4 | basic region-leucine zipper | Signal transduction and DNA binding |
| WRKY | 15 | 4 | 11 | WRKY TFs | Defense responses and DNA binding |
| AP2/ERF | 8 | 3 | 5 | Ethylene responsive TFs | Organ development and stress response |
| MADS | 3 | 1 | 2 | MADS-box TFs | Organ development and signal transduction |
| C2C2 | 9 | 4 | 5 | C2C2 zinc finger protein | Development and DNA binding |
| C2H2 | 15 | 3 | 12 | C2H2 zinc finger protein | Cell division and differentiation |
| GARP | 7 | 3 | 4 | Myb-like DNA-binding domain | DNA-binding and signal transduction |
| SBP | 2 | 0 | 2 | squamosa promoter binding | Signal transduction and DNA binding |
| NAC | 8 | 3 | 5 | NAC TFs | Signal transduction and DNA binding |
| GRAS | 2 | 0 | 2 | GRAS TFs | Signal transduction and DNA binding |
| AUX/IAA | 4 | 2 | 4 | Auxin-responsive protein | Signal transduction and DNA binding |
| HB-HD-ZIP | 7 | 2 | 5 | Homeobox-leucine zipper protein | Plant development and signal transduction |
| C3H | 14 | 7 | 7 | Zinc finger CCCH domain | Plant development and RNA-binding, |
| Other TFs | 132 | 50 | 82 |  |  |
| In total | 273 | 104 | 169 |  |  |

P, Bronze fennel; G, Florence fennel. Differentially expressed genes were identified by FDR ≤ 0.001 and absolute value of log_2_ ratio ≥ 1.
