# Supplementary material for: Integrated Transcriptomic and Metabolomic Analyses Reveal the Mechanisms Underlying Anthocyanin Coloration and Aroma Formation in Purple Fennel
Source: Front Nutr. 2022 Apr 27;9:875360. doi: 10.3389/fnut.2022.875360 (PMC9093692; doi:10.3389/fnut.2022.875360)
Supplement: Supplementary file 7 [file Data_Sheet_1.doc]

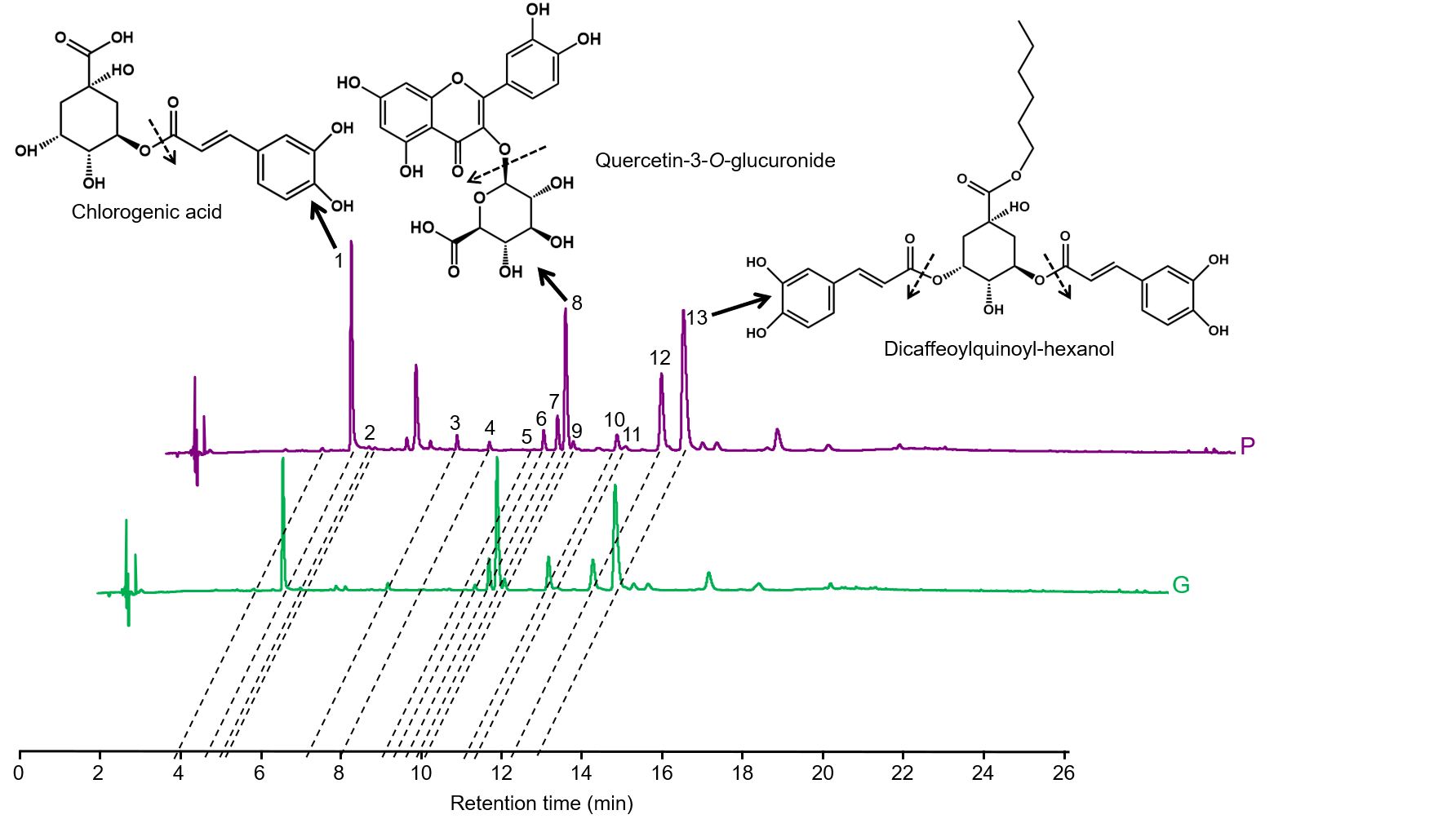


**Supplementary Figure 1.** The major phenolic compounds except anthocyanins identified in fennel. The main constitutes in fennel are depicted as component 1 to 13 on the HPLC-diode-array-detection (DAD) trace (330-nm detection). Flavonol derivates display a structural homology where the common building blocks are a flavonol chromophore (kaempferol and quercetin), and sugars. The key differentiation is the substitution patterns of saccharide moiety bound at the position of chromophore. Major cleavage sites of chlorogenic acid, quercetin 3-O-glucuronide and the deduced component dicaffeoylquinoyl-hexanol verified by mass spectrometry were indicated by dashed arrow, respectively.

]

**
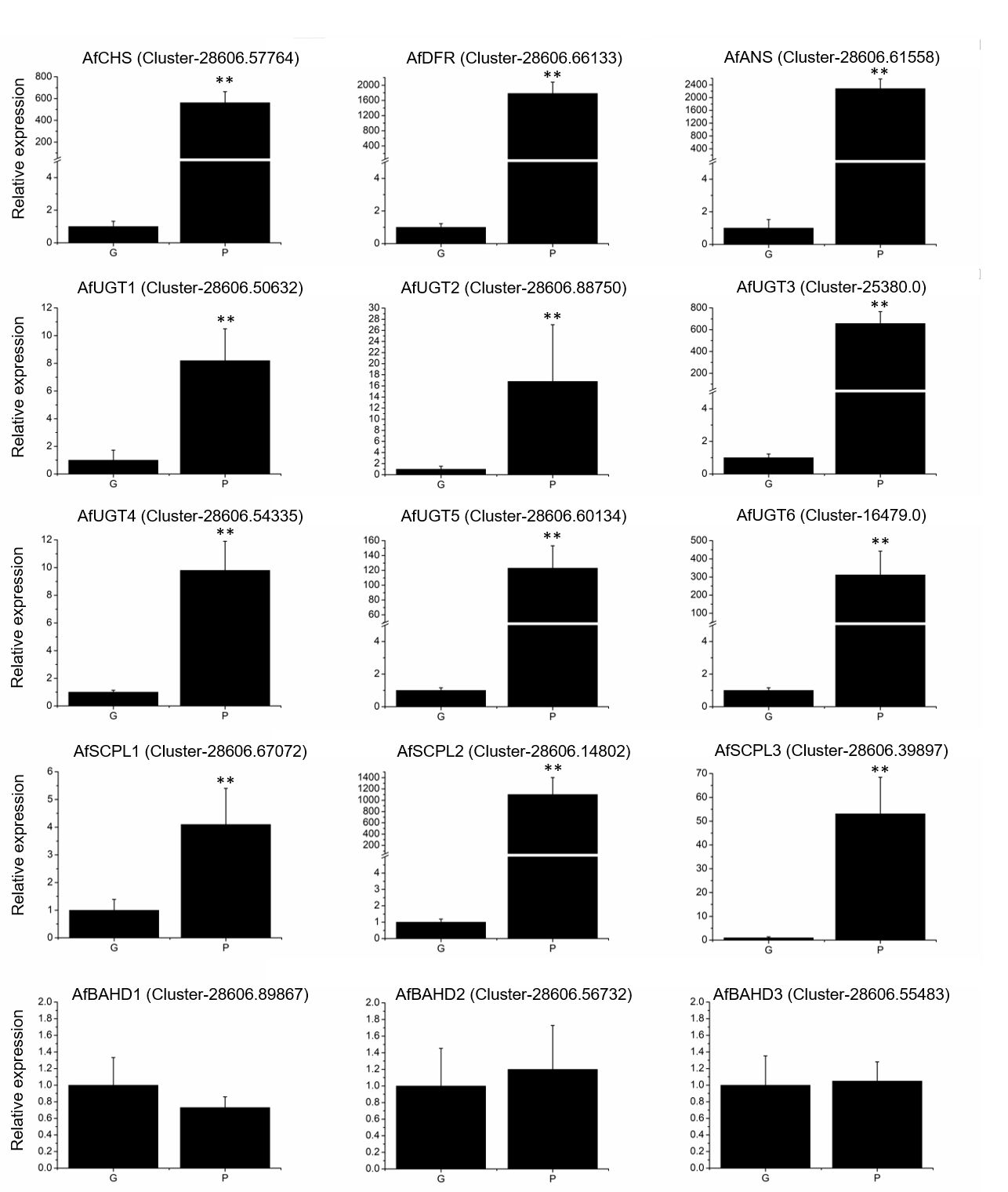
**

**Supplementary Figure 2.** Expression analysis of the genes associated with anthocyanin biosynthesis and modification in fennel. Partial selected anthocyanin structural genes, glycosyltransferase genes, serine carboxypeptidase-like acyltransferase genes, and BAHD acyltransferase genes were indicated. G and P indicate the green and purple fennel variety, respectively.


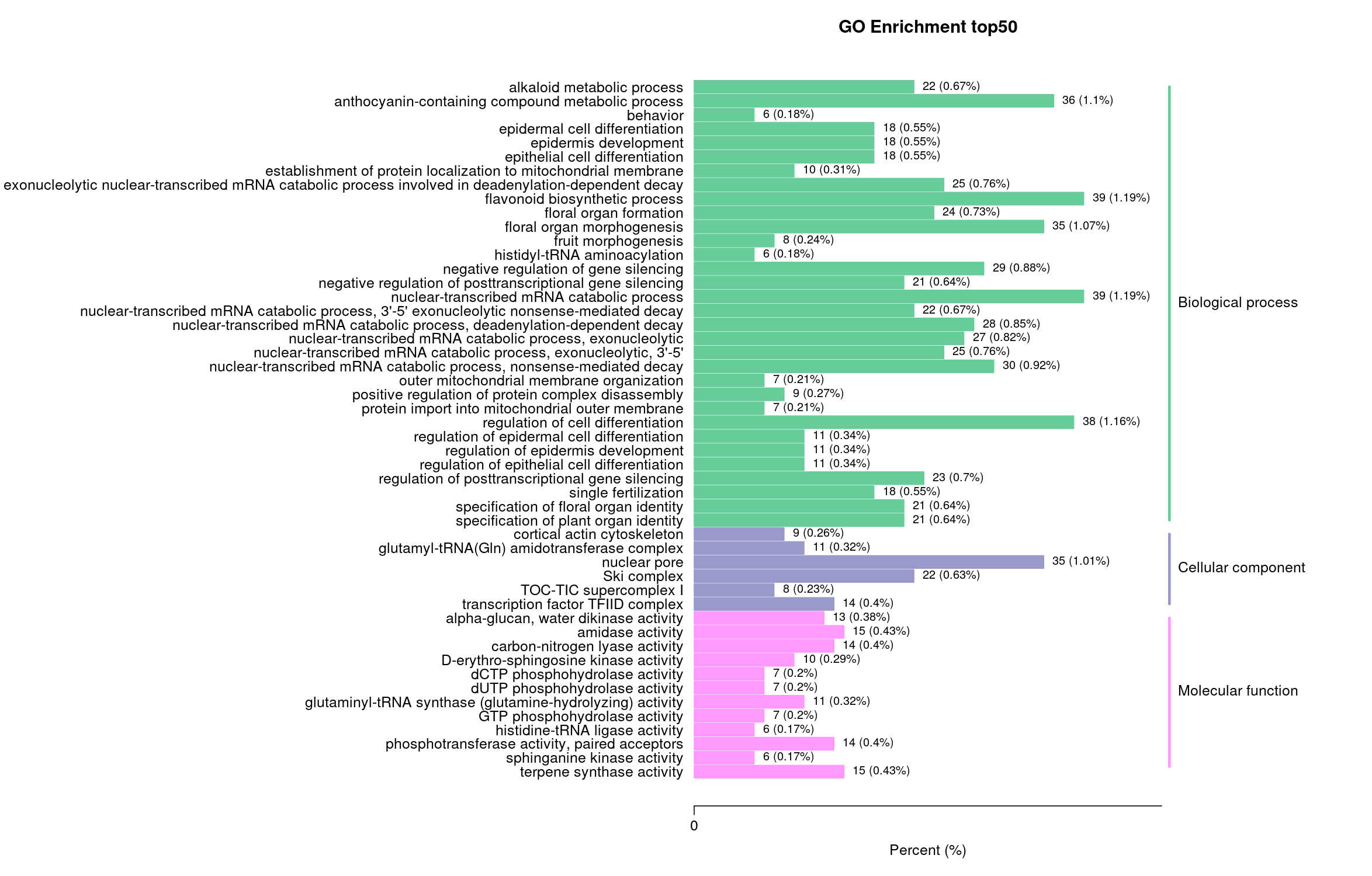


**Supplementary Figure 3.** Gene ontology (GO) functional classification of DEGs in fennel.


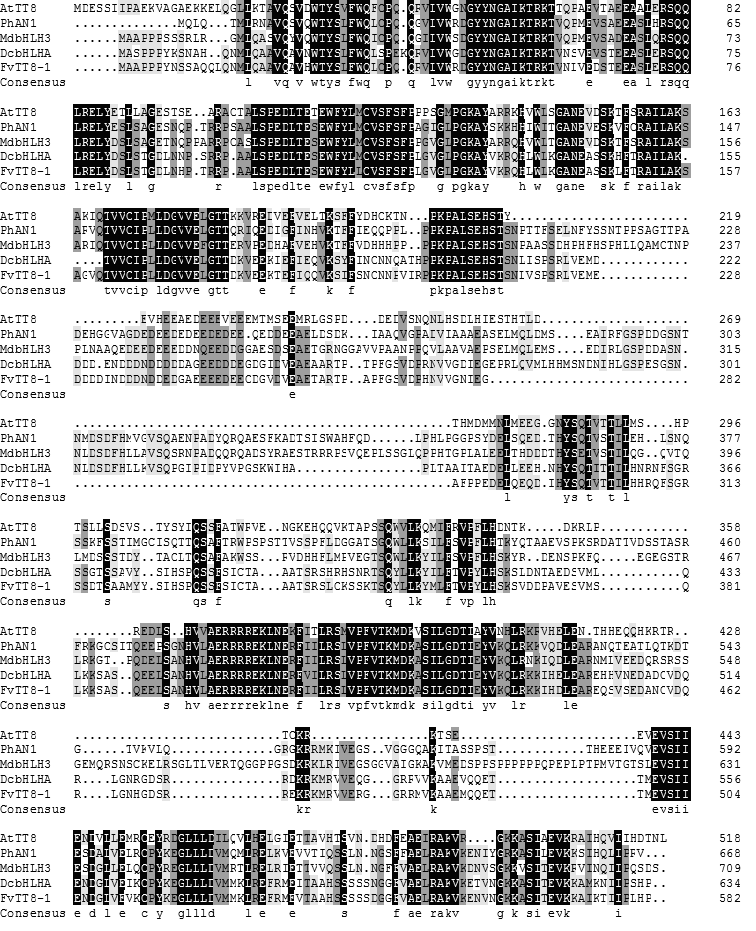


**MYB interaction region**

**bHLH domain**

**Supplementary Figure 4.** A multiple sequence alignment of AfTT8 with bHLH homologues from *Petunia x hybrida* (PhAn1), *Malus domestica* (MdbHLH3), *Daucus carota* (DcbHLHA) and *Arabidopsis thaliana* (AtTT8). The specific residues for MYB interaction domain were underlined and a specific bHLH domain was framed.


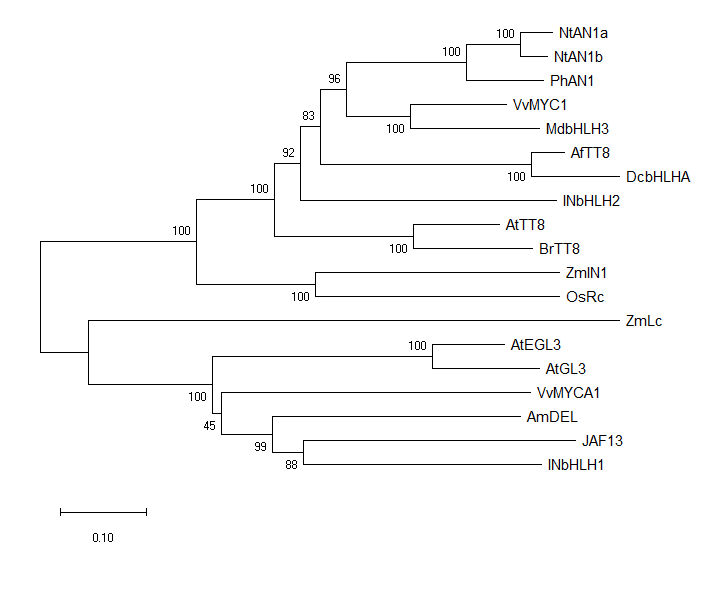


**Supplementary Figure** **5.** Phylogenetic reconstruction of AfTT8 with selected bHLHs using neighbor-joining method with MEGA Ⅹ software. A minimum evolutionary phylogeny test and 1,000 bootstrap replicates were chosen for analysis. The scale bar represents 0.1 substitutions persite. The bHLH proteins, their respective plant species, and GenBank accession numbers are shown as follows: SlTT8, *Solanum lycopersicon* (Solyc09g065100.1.1); BrTT8, *Brassica rapa* (AIP94025); PhAn1, *Petunia x hybrid* (AAG25927); InbHLH1, *Ipomoea nil* (BAE94393); InbHLH2, *Ipomoea nil* (BAE94394); VvMYCA1, *Vitis vinifera* (ABM92332); VvMYC1, (ACC68685); CsMYC2, *Citus sinensis* (ABR68793); PfMYC-RP, *Perilla frutescens* (BAA75513); F3G1, *Perilla frutescens* (BAC56998); AmDelila, *Antirrhinum majus* (AAA32663); PhJAF13 (AAC39455); AtGL3, *Arabidopsis thaliana* (NP_680372); AtEGL3 (NP_176552); AtTT8 (CAC14865); ZmIN1 (AAB03841); ZmLc (NP_001105339); OsRc, *Oryza sativa* (BAF42668); PsbHLH, *Pisum sativum* (ADO13282); MdbHLH3 (ADL36597); NtAN1a (AEE99257); NtAN1b (AEE99258).


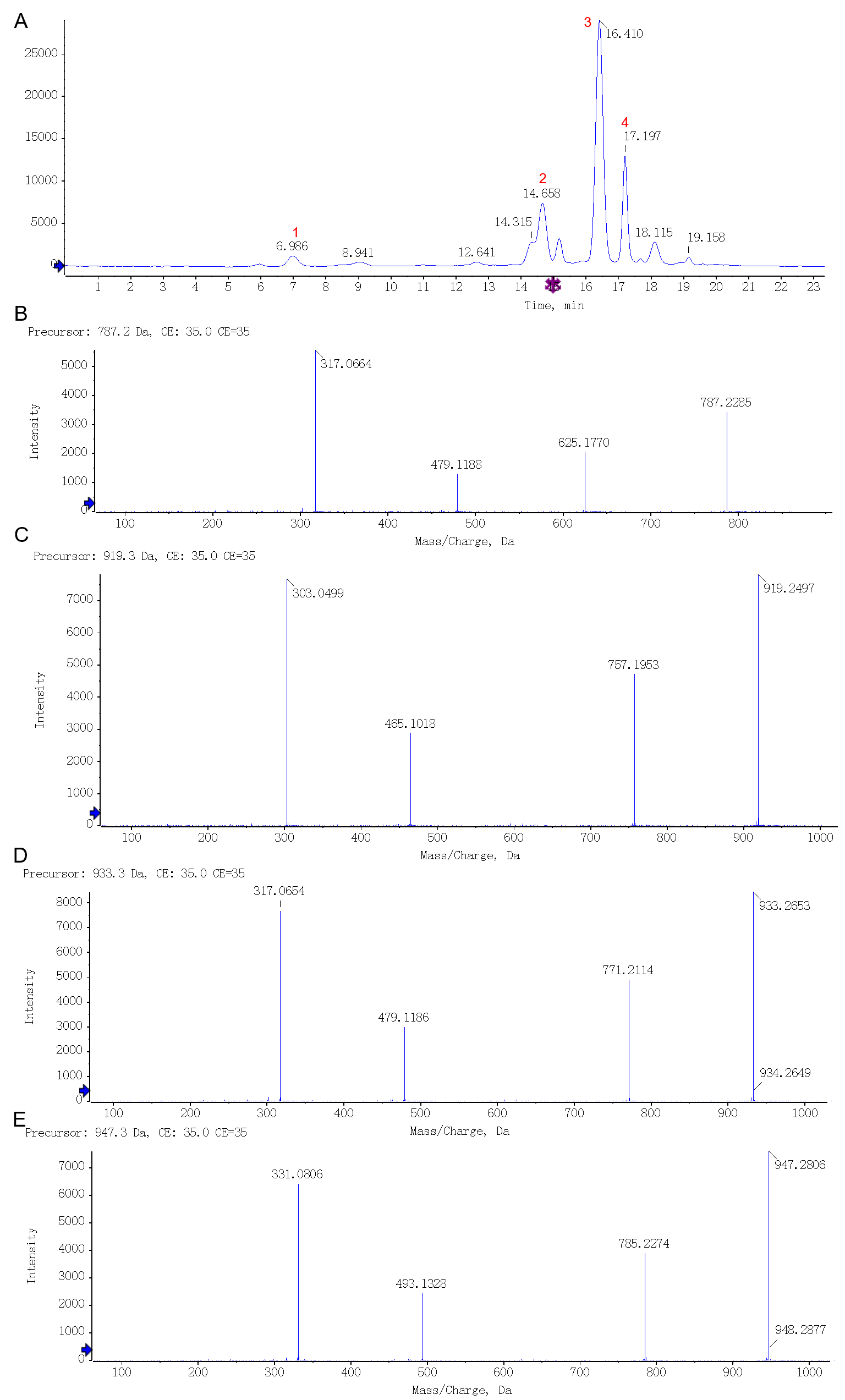


**Supplementary Figure** **6.**  Identification of the anthocyanins extracted from transgenic tomato fruits. (A) UHPLC profile of the anthocyanins extracted from transgenic tomato fruits. Peak number refers to the individual anthocyanin, respectively. (B) MS^2^ spectra of petunidin 3-rutinoside-5-glucoside corresponding to peak 1 in the chromatogram of figure 6 A. (B) MS^2^ spectra of delphinidin 3-(trans-coumaroyl)-rutinoside-5-glucoside corresponding to peak 2. (C) MS^2^ spectra of petunidin 3-(trans-coumaroyl)-rutinoside-5-glucoside corresponding to peak 3. (D) MS^2^ spectra of malvnidin 3-(trans-coumaroyl)-rutinoside-5-glucoside corresponding to peak 4 in figure A.

**
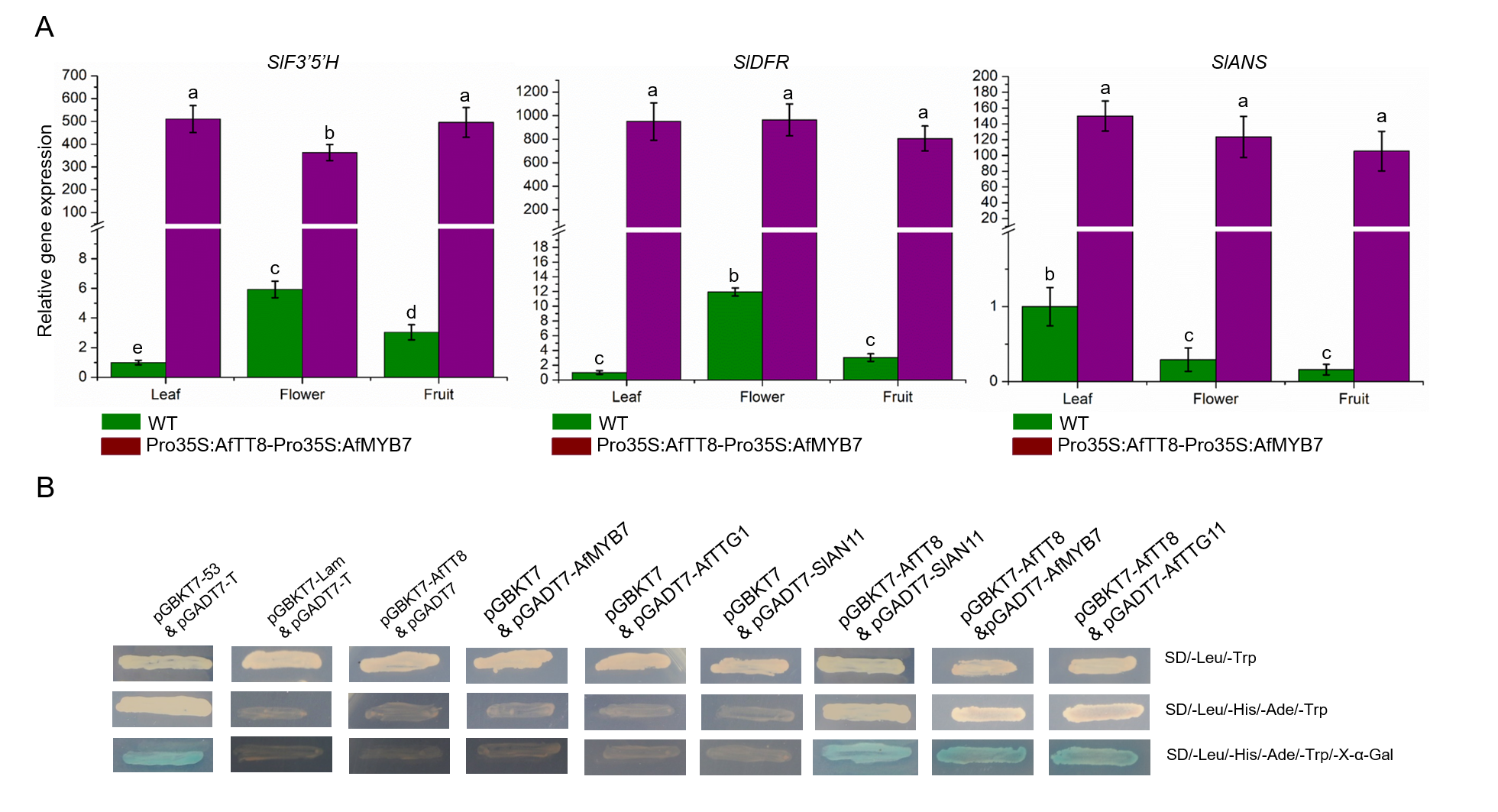
Supplementary Figure** **7.** A functional MBW complex regulating anthocyanin accumulation through activating structural genes at transcriptional level in tomato. (A) transcripts of several anthocyanin structural genes in various tissues of WT and transgenic tomato plants. The longitudinal axis shows relative gene expression and the different letters represents significance (P < 0.05). (B) AfTT8 interacts efficiently with AfMYB7, SlAN11, and AfTTG1, respectively.


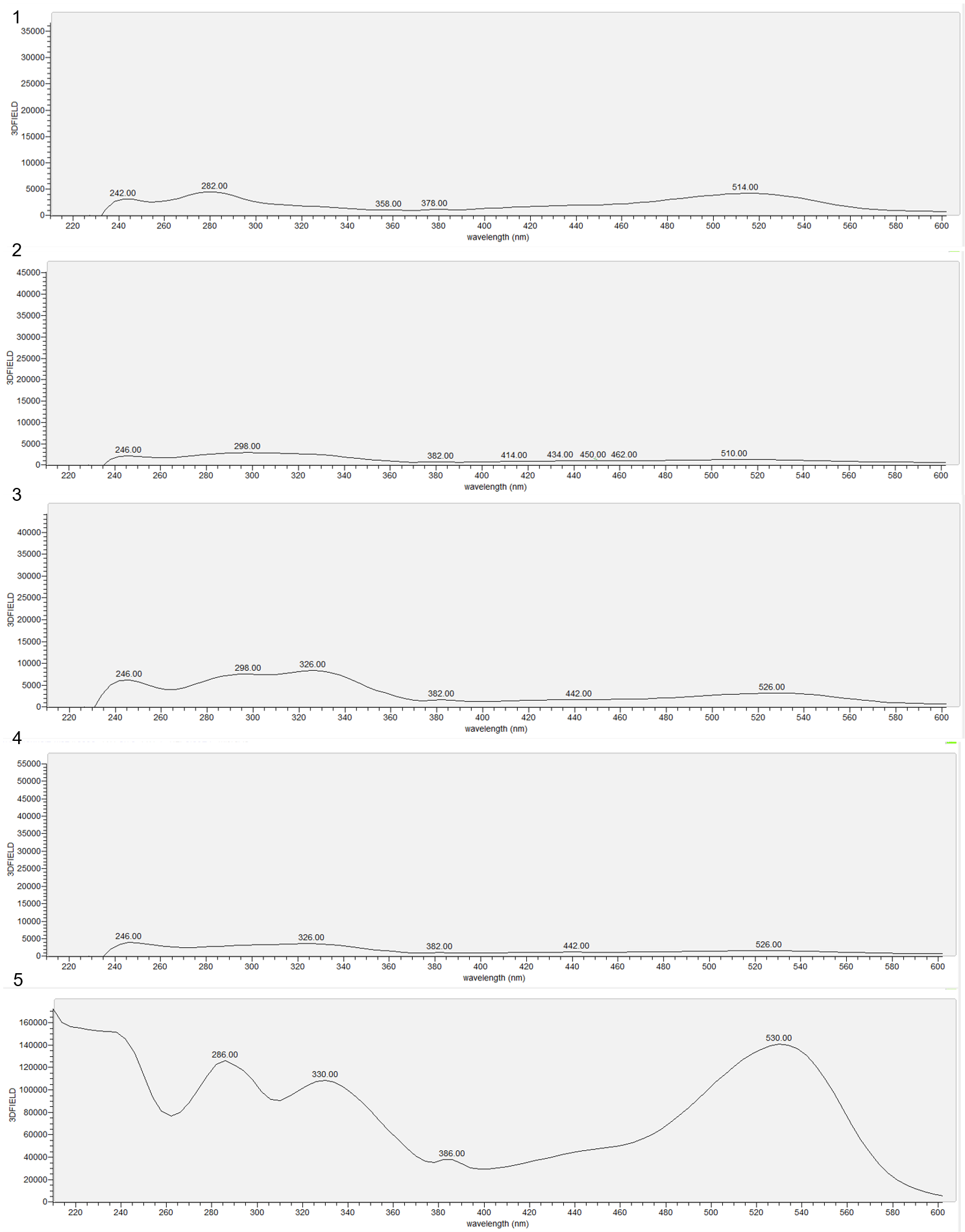


**Supplementary Figure** **8.**  UV-vis spectra of the identified anthocyanins in purple fennel depicted as component 1 to 5 in chromatogram of figure 1 or table 1.


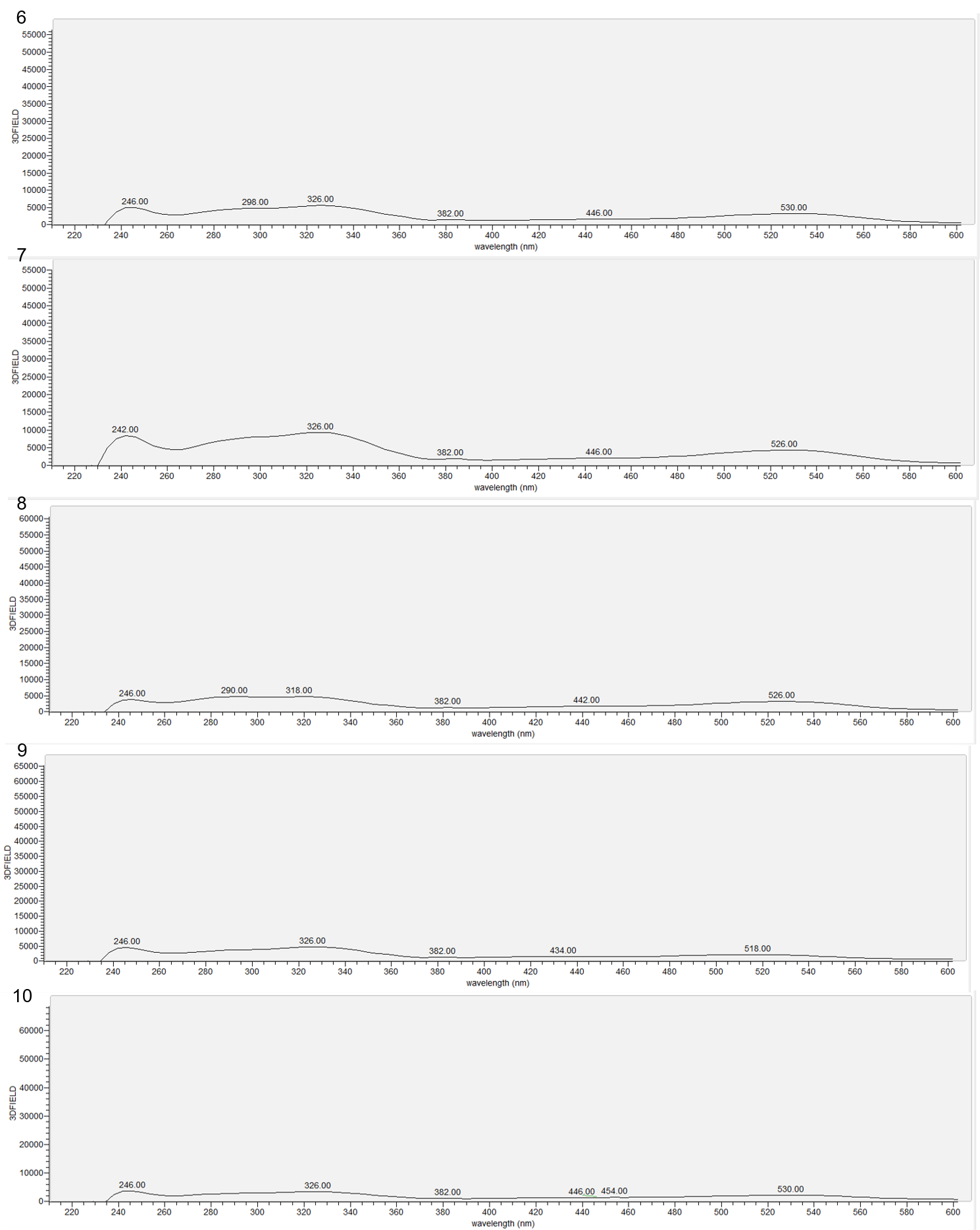


**Supplementary Figure** **9.**  UV-vis spectra of the identified anthocyanins in purple fennel depicted as component 6 to 10 in chromatogram of figure 1 or table 1.


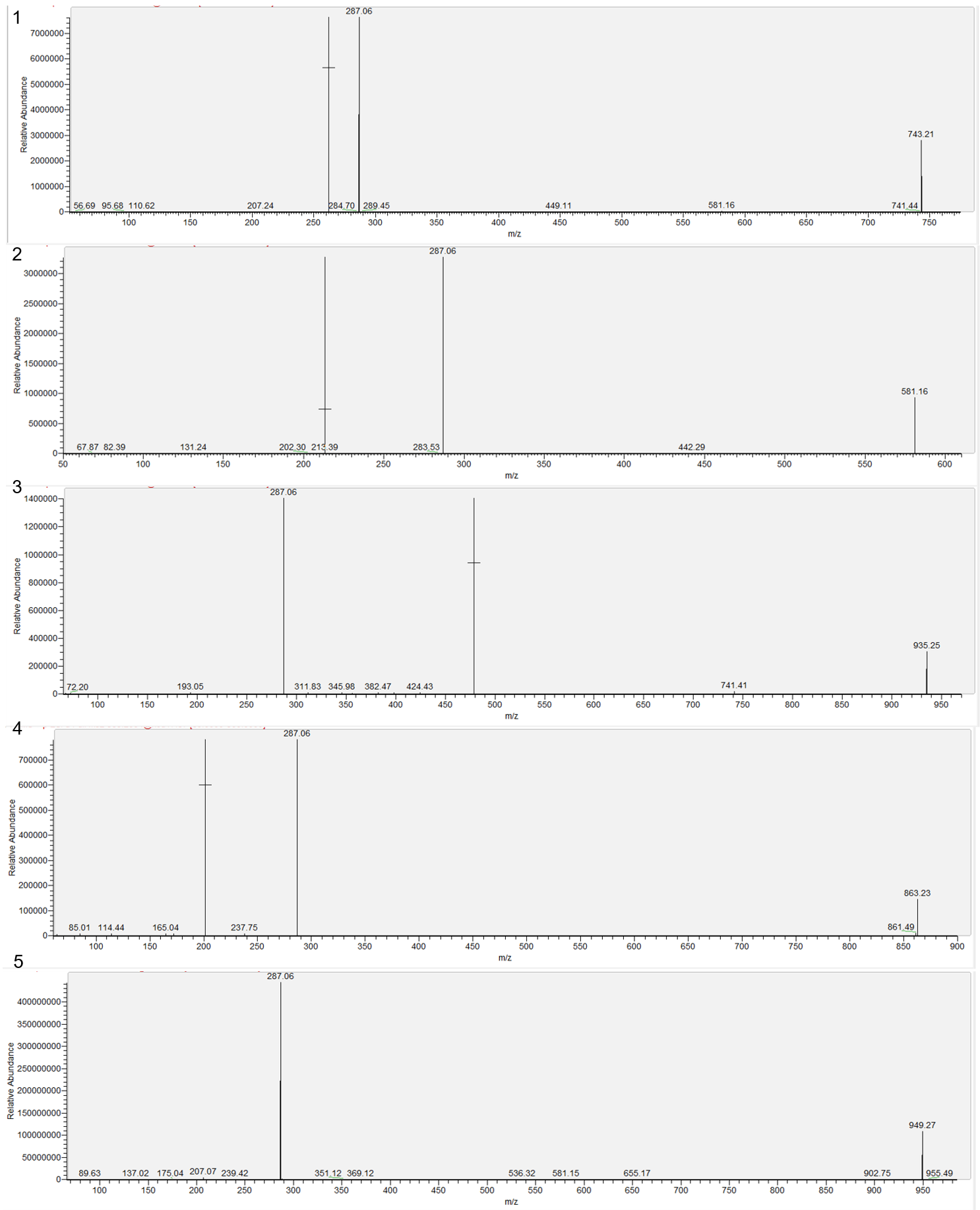


**Supplementary Figure** **10.**  MS^2^ spectra of the identified anthocyanins in purple fennel depicted as component 1 to 5 in chromatogram of figure 1 or table 1.


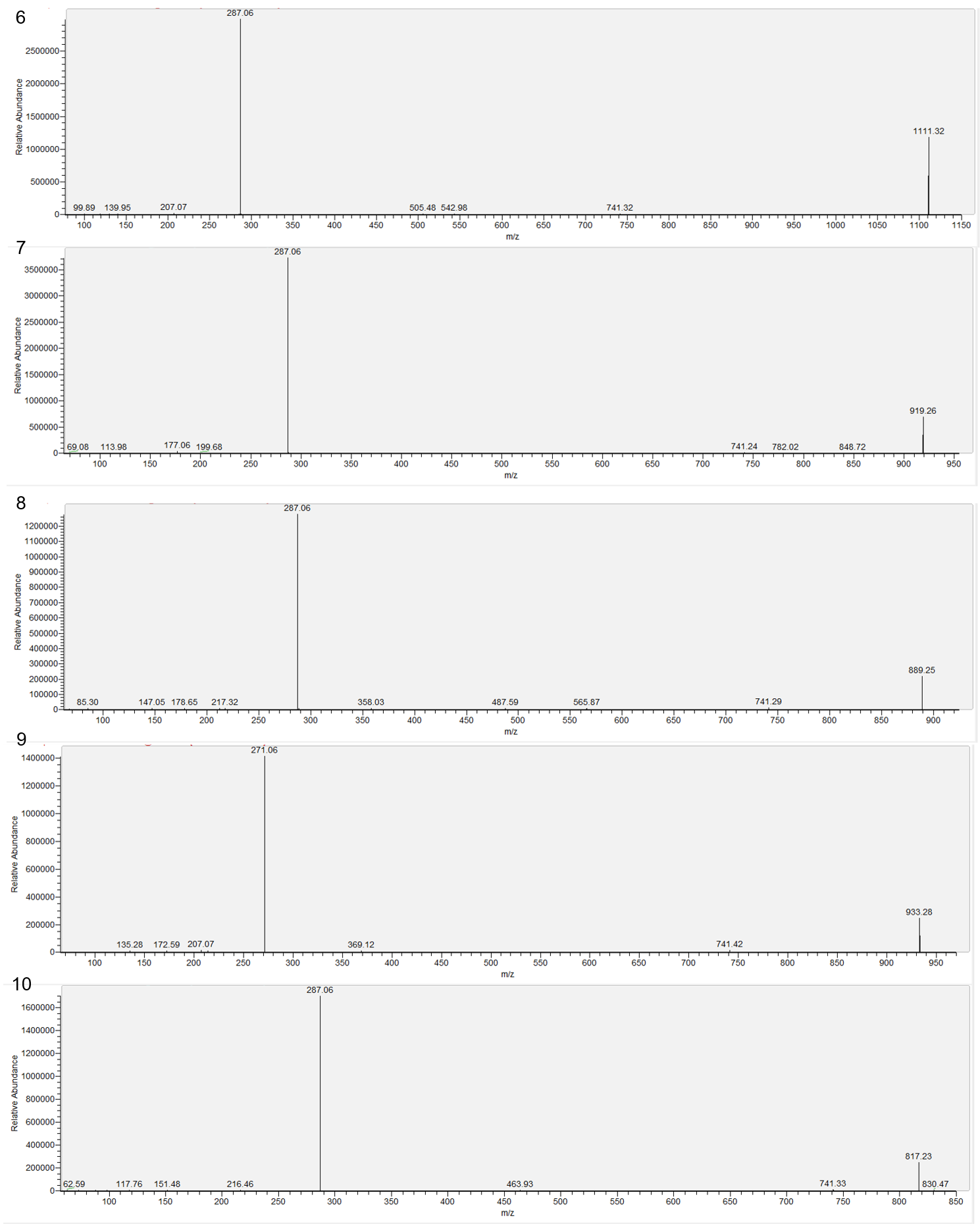


**Supplementary Figure** **11.**  MS^2^ spectra of the identified anthocyanins in purple fennel depicted as component 6 to 10 in chromatogram of figure 1 or table 1.


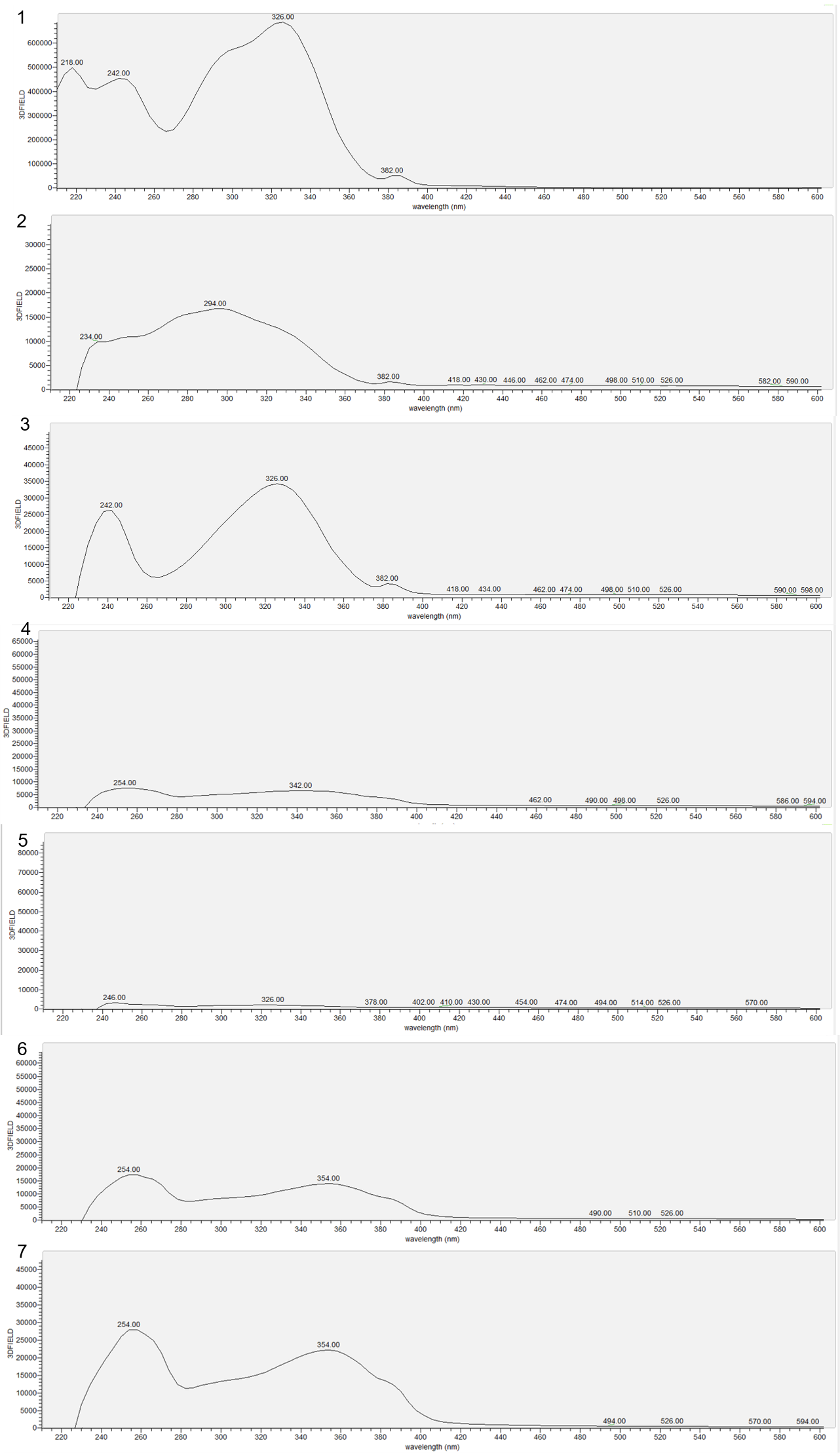


**Supplementary Figure** **12.**  UV-vis spectra of the identified phenolic compounds in fennel depicted as component 1 to 7 in chromatogram of supplementary figure 1 or table 2.


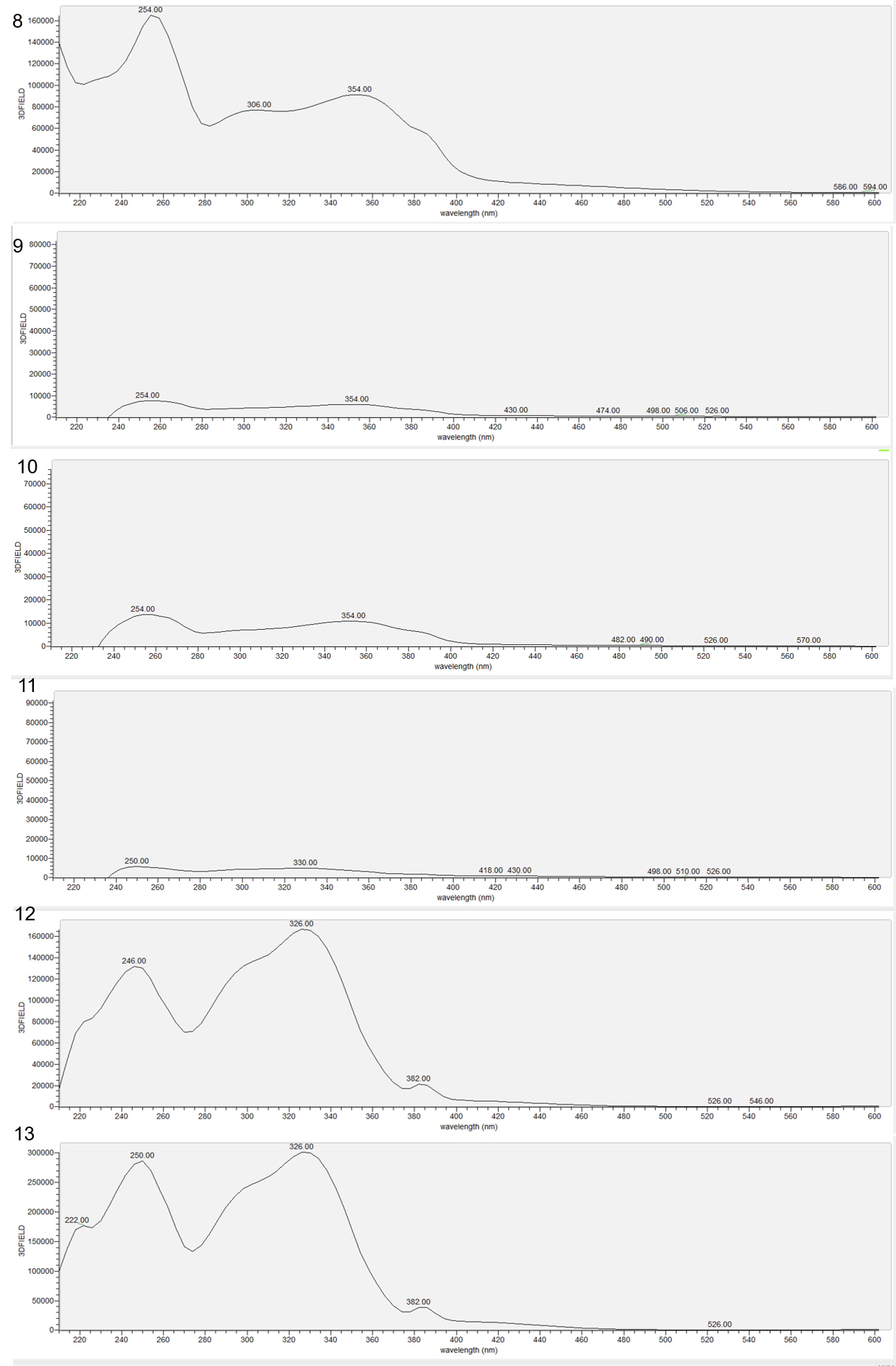


**Supplementary Figure** **13.**  UV-vis spectra of the identified phenolic compounds in fennel depicted as component 8 to 13 in chromatogram of supplementary figure 1 or table 2.


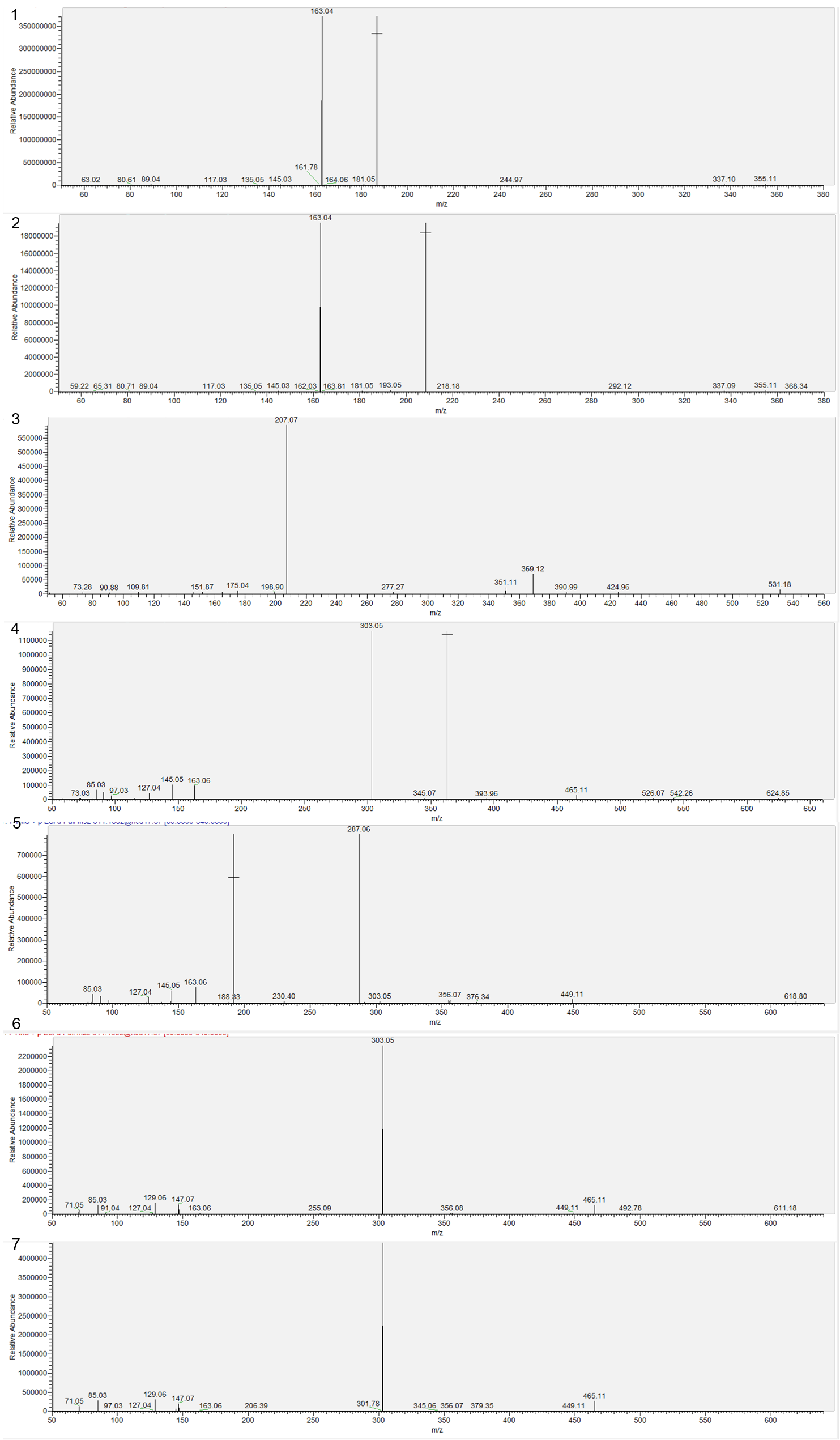


**Supplementary Figure** **14.**  MS^2^ spectra of the identified phenolic compounds in fennel depicted as component 1 to 7 in chromatogram of supplementary figure 1 or table 2.


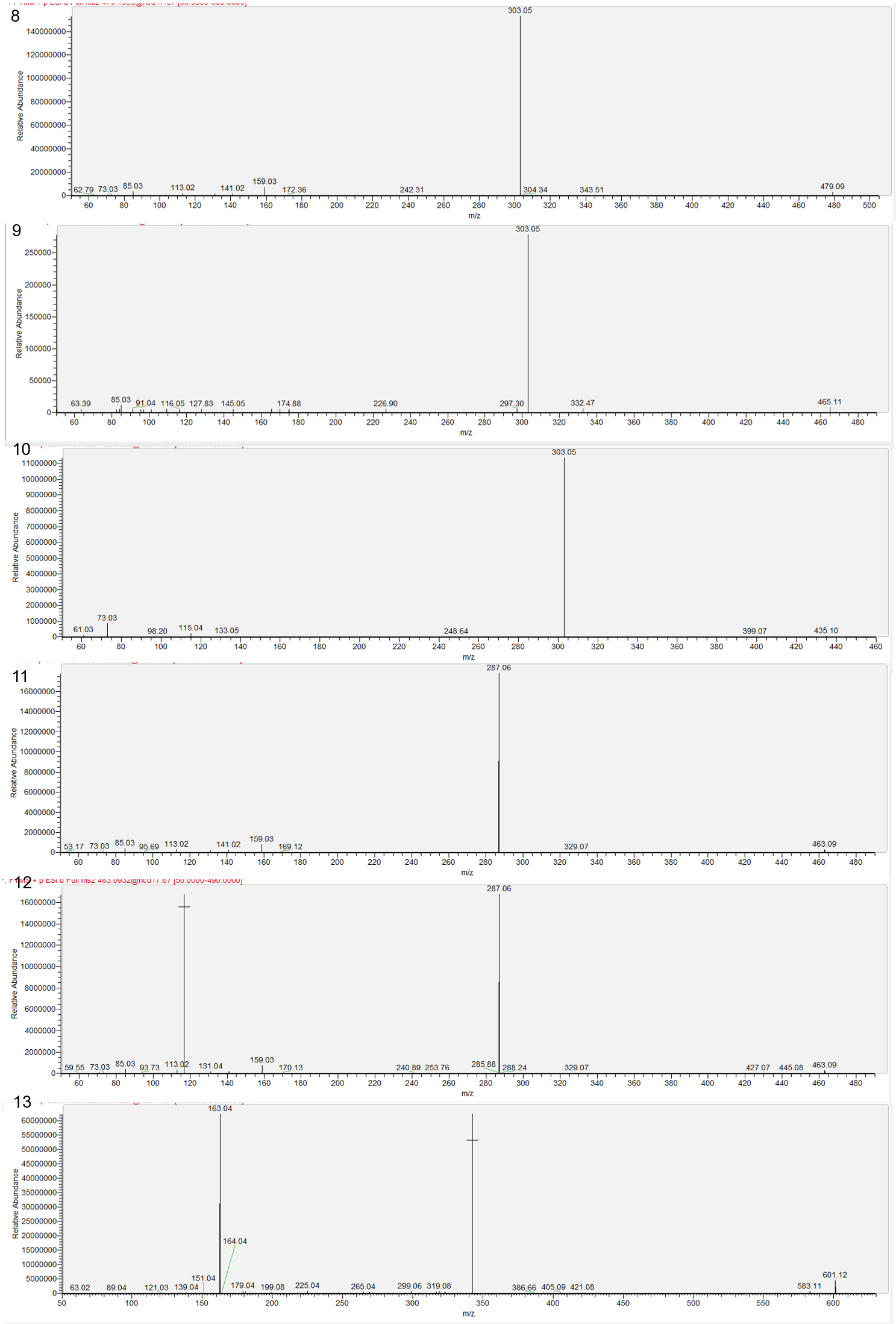


**Supplementary Figure** **15.**  MS^2^ spectra of the identified phenolic compounds in fennel depicted as component 8 to 13 in chromatogram of supplementary figure 1 or table 2.
